# Precision-Elastic Persistent Stochastic Execution for Quantum Circuit Simulation

**DOI:** 10.3390/e28080862

**Published:** 2026-08-01

**Authors:** Naoya Onizawa, Martin Lukac, Shinobu Nagayama, Takahiro Hanyu

**Affiliations:** 1 Research Institute of Electrical Communication, Tohoku University, Sendai 980-8577, Japan; 2 Graduate School of Information Sciences, Hiroshima City University, Hiroshima 731-3194, Japan; malu@hiroshima-cu.ac.jp (M.L.);

**Keywords:** quantum circuit simulation, stochastic computing, integral stochastic computing, approximate computing, precision elasticity, QASMBench

## Abstract

Quantum circuit simulation is usually evaluated through final numerical accuracy, while the dynamics of stochastic execution itself are less explicitly characterized. This work presents a precision-elastic persistent stochastic execution framework based on integral stochastic computing (ISC), where execution behavior is controlled by stream length *N* and ISC multiplicity *m*, where m represents the number of aggregated stochastic sub-streams per cycle (standard SC corresponds to *m* = 1). We focus on correlation-sensitive propagation under persistent reuse, and show that this regime produces circuit-dependent stochastic behavior and execution uncertainty patterns that are not captured by stage-wise re-encoded execution alone. To characterize this behavior, we use high-*m* tail descriptors, including the circuit-dependent coefficient $\gamma_{c}$, as compact indicators of persistent stochastic sensitivity. Across benchmark circuits, persistent execution exhibits reproducible tail regimes and structured cross-circuit variability, while deterministic reduced-precision baselines are used only as trend-consistency references. We further demonstrate adaptive stochastic precision scheduling, where circuit-dependent (N,m) settings satisfy a target fidelity with reduced stochastic workload. These results position persistent ISC as a configurable stochastic execution framework for analyzing execution-induced uncertainty propagation and correlation-sensitive behavior in quantum circuits.

## 1. Introduction

Quantum circuit simulation remains a core tool for algorithm design, verification, and system-level co-design. In current practice, most simulators rely on deterministic numerical kernels, including statevector and tensor-network approaches [1,2,3], implemented in software stacks such as qsim, Qiskit Aer, and cuQuantum [4,5,6]. Benchmark suites such as QASMBench provide standardized workloads for controlled evaluation [7]. While these methods provide high accuracy and reproducibility, their numerical precision is typically fixed by design and they primarily emphasize final numerical outputs, offering limited visibility into stochastic information propagation and execution uncertainty accumulation under correlation-retaining conditions.

In addition, most existing quantum simulators operate on platforms optimized for fixed numerical precision, such as floating-point execution on CPUs/GPUs [4,5,6] and fixed-point-oriented implementations [8,9]. However, these approaches do not directly expose precision as a runtime control variable for studying execution variability and uncertainty propagation. As a result, precision adaptation often requires non-trivial reconfiguration of numerical kernels rather than direct control in execution space. From this perspective, stochastic computing provides a useful foundation, because its arithmetic naturally exposes precision as a tunable resource.

To explore this perspective, this paper adopts stochastic execution as an analytical and computational framework. In stochastic computing (SC), values are represented by bitstream statistics, enabling precision control through stream length *N* [10]. Integral stochastic computing (ISC) extends this paradigm with the number of aggregated stochastic sub-streams *m*, providing two-dimensional precision control [11]. Prior work has shown that correlation between stochastic streams significantly affects arithmetic accuracy [12,13], but existing analyses are largely confined to local arithmetic structures. In contrast, this work focuses on cross-layer propagation dynamics, examining how stochastic dependence behaves when it is either reset between gates (re-encoded ISC) or preserved across gates (persistent ISC).

Within this framework, we define three stochastic execution regimes: SC, ISC-RE, and ISC-PS. Re-encoded execution approximately restores stage-wise independence, whereas persistent execution carries stochastic dependence across layers, potentially amplifying circuit-structure effects and uncertainty accumulation. Unlike conventional numerical methods, the proposed framework enables continuous and runtime-adjustable control of the accuracy–cost trade-off through (N,m), providing a flexible paradigm for analyzing execution-induced behavior. This positioning is distinct from other stochastic approaches in quantum simulation: noise-aware frameworks model physical channels and hardware error processes [14,15], while Monte Carlo methods use randomness for estimator sampling [16,17]. In contrast, stochasticity in this work arises from numerical representation and arithmetic, and the resulting behavior reflects execution-induced dependence rather than physical decoherence. This perspective complements deterministic reduced-precision methods [18,19,20] and connects to structured stochastic-dynamics viewpoints in probabilistic computing [21].

We evaluate these regimes using multi-seed *m*-sweeps on QASMBench circuits (c1–c12). Persistent execution exhibits reproducible tail behavior together with circuit-dependent sensitivity patterns, summarized using two descriptors: a high-*m* tail slope and a circuit-dependent tail coefficient $\gamma_{c}$. Together, these descriptors capture persistent stochastic sensitivity and correlation-sensitive execution dynamics.

The contributions of this paper are as follows:

- Precision-elastic stochastic simulation framework.We introduce ISC-PS as a stochastic execution framework with explicit and tunable control over accuracy and computational cost through (N,m).
- Regime-level execution analysis. We formalize persistent execution as a distinct execution regime and compare it directly against re-encoded ISC under matched settings.
- Circuit-dependent behavior characterization. We propose tail descriptors, including $\gamma_{c}$, to quantify correlation-sensitive persistent behavior across circuits.
- Target-fidelity adaptive precision scheduling. We demonstrate that the proposed two-parameter precision space can be used operationally by selecting circuit-dependent (N,m) pairs that satisfy a prescribed fidelity target with reduced stochastic cost.
- Structure-linked interpretation. We demonstrate that circuit-structure descriptors provide explanatory signal for persistent amplification behavior under stochastic execution.

The remainder of this paper is organized as follows. Section 2 reviews related work. Section 3 introduces preliminaries, and Section 4 presents the stochastic execution framework. Section 5 analyzes propagation behavior and defines the descriptors used in this study. Section 6 describes the experimental methodology, Section 7 reports results, Section 8 discusses implications, and Section 9 concludes the paper.

## 2. Related Work

### 2.1. Quantum Simulation and Precision Control

Quantum circuit simulation is typically performed using deterministic numerical kernels, including statevector and tensor-network approaches [1,2,3]. In practice, these simulators rely on floating-point arithmetic as the standard baseline due to its dynamic range and numerical stability. Recent work has further explored performance improvements through hardware acceleration and reduced-precision variants, such as mixed-precision implementations [4,5,6]. These approaches primarily study the trade-off between computational efficiency and numerical accuracy within deterministic arithmetic, where precision is typically fixed by design.

Recent quantum-computing research has also increasingly considered practical algorithm execution under finite resource, performance, robustness, and scalability constraints. Hybrid quantum-classical frameworks provide one example of this execution-oriented direction; for instance, Zheng et al. proposed a Grover adaptive search-based hybrid Benders decomposition method for mixed-integer linear programs that integrates a quantum search procedure with a classical decomposition workflow while analyzing execution efficiency, stability, and scalability [22]. This direction is complementary to the present work: rather than developing a hybrid optimization algorithm, we study stochastic numerical execution, persistent dependence propagation, and resource-aware precision control for quantum circuit simulation.

Recent work has explored stochastic approaches for quantum circuit simulation, including stochastic optical simulation and decision-diagram-based stochastic simulation frameworks [15,23]. These approaches use stochasticity at the simulator/modeling level (e.g., optical-process or noise-related stochastic modeling), whereas our work introduces stochasticity through numerical representation and arithmetic under persistent execution, enabling analysis of cross-layer correlation accumulation.

### 2.2. Stochastic Computing and Correlation

Stochastic computing represents values using bitstream statistics and enables precision control via stream length [10]. Integral stochastic computing extends this model with an additional aggregation parameter *m*, introducing a second degree of freedom for precision control [11]. A key issue in SC is correlation, which significantly affects arithmetic accuracy [12,13]. Existing work has mainly analyzed correlation at the arithmetic-block or circuit level, focusing on local structures and correlation engineering. However, the use of stochastic representations as a configurable simulation framework with explicit control over accuracy and computational cost remains relatively unexplored.

### 2.3. Cross-Layer Error Propagation

Error propagation across layers has been studied in deterministic reduced-precision settings, such as fixed-point and quantization-aware computation [18,19,20]. These approaches model how rounding and quantization errors accumulate across layers, providing insights into stability and accuracy degradation. However, they do not consider dependence induced by stochastic representations, where correlations may propagate across stages depending on execution mode.

### 2.4. Position of This Work

This work introduces an integral stochastic computing framework for quantum circuit simulation, centered on stochastic execution modes including ISC-RE and ISC-PS, with explicit control over the accuracy–cost trade-off through two parameters, *N* and *m*. Unlike deterministic representations, where precision is statically determined, the proposed framework enables continuous and runtime-adjustable precision through stochastic execution.

Within this framework, we consider both re-encoded (ISC-RE) and persistent (ISC-PS) execution modes. Persistent execution preserves cross-layer dependence, which leads to circuit-dependent behavior that differs from conventional deterministic or re-encoded stochastic models. In this paper, such behavior is treated as a characteristic of the framework rather than the sole objective.

The proposed approach complements floating-point and fixed-point simulation by adding an explicit stochastic accuracy–cost control axis. In particular, ISC enables explicit and tunable trade-offs between computational cost and numerical fidelity, which are difficult to achieve in conventional fixed-precision arithmetic.

Table 1 summarizes the qualitative differences between prior approaches and this work. Table 2 summarizes the main terms, parameters, and descriptors used throughout the paper.

## 3. Preliminaries

### 3.1. Quantum State Representation

An *n*-qubit pure state is represented as

(1) $$|\psi \rangle =\sum_{i=0}^{2^{n}-1}\psi_{i}|i\rangle ,\;\sum_{i=0}^{2^{n}-1}{\left|\psi_{i}\right|}^{2}=1.$$

**Figure 1:**
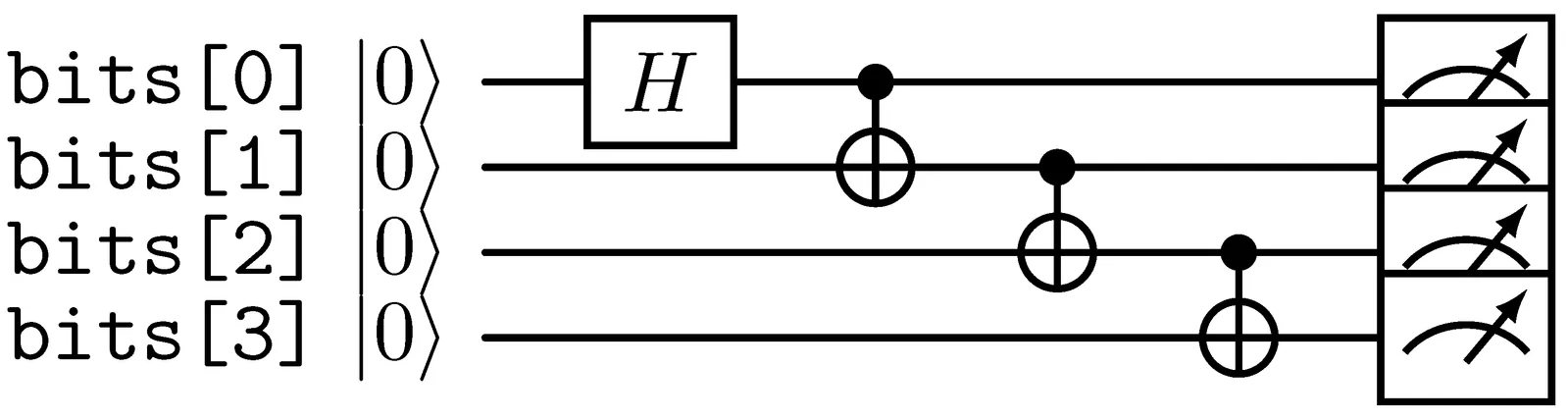
QASMBench circuit c1 (cat_state_n4, n=4), showing GHZ-like state preparation followed by measurement. The circuit corresponds to a Hadamard on the first qubit, a CNOT chain across all four qubits, and final measurement on each qubit.

Each amplitude $\psi_{i}=a_{i}+jb_{i}$ evolves under unitary gates while preserving global normalization. For circuit c1 in Figure 1, the statevector dimension is $2^{4}=16$, and each gate corresponds to a sparse structured linear transform on that vector. Gate-wise state evolution follows

(2) $$\psi_{k+1}=U_{k}\psi_{k},$$

where $U_{k}\in C^{2^{n}\times 2^{n}}$ (here, $U_{k}\in C^{16\times 16}$). Here, a layer refers to one gate-application/state-update stage in the simulation flow, while *L* denotes the total number of such layers, i.e., the circuit depth. In our SC/ISC implementation, the corresponding complex matvec is realized by stochastic primitives: bipolar multiplication (XNOR), stochastic addition (MUX-based weighted add), and, for ISC, integer-range accumulation controlled by *m*.

#### Circuit Model and Primitive Gates (QASMBench c1–c5)

We illustrate definitions using representative QASMBench circuits c1–c5: c1 (cat_state_n4), c2 (adder_n4), c3 (basis_change_n3), c4 (qft_n4), and c5 (lpn_n5) [7]. The full persistent-regime evaluation later spans c1–c12. In the circuit diagram, *H* denotes the Hadamard gate, with 

(3) $$H|0\rangle =\frac{|0\rangle +|1\rangle }{\sqrt{2}},\;H|1\rangle =\frac{|0\rangle -|1\rangle }{\sqrt{2}}.$$

The controlled-NOT (CX/CNOT) is represented by a control dot and target ⊕, and acts as

(4) $$\mathrm{CX}|c,t\rangle =|c,\;t⊕c\rangle .$$

Measurement symbols (meter icons) indicate projection to classical bits at readout. These primitive choices and their depth-dependent composition influence intermediate amplitude magnitudes and correlation strength in persistent execution, which directly affects later RE–PS error behavior.

### 3.2. Bipolar Stochastic Encoding

In bipolar stochastic computing, a scalar x∈[−1,1] is encoded as a Bernoulli bitstream ${\left\{s_{k}\right\}}_{k=1}^{N}$ with $s_{k}\in \left\{-1,+1\right\}$ such that

(5) $$E[s_{k}]=x.$$

The stochastic estimator is

(6) $$\hat{x}=\frac{1}{N}\sum_{k=1}^{N}s_{k},$$

with variance

(7) $$\mathrm{Var}\left[\hat{x}\right]=\frac{1-x^{2}}{N}.$$

Hence, the SC estimator accuracy improves as O(1/N) under independent sampling.

### 3.3. Stochastic Arithmetic Primitives

#### 3.3.1. Multiplication

Let *a* and *b* be encoded as independent bipolar bitstreams $\left\{a_{k}\right\}$ and $\left\{b_{k}\right\}$. Multiplication is implemented via bitwise XNOR:

(8) $$c_{k}=a_{k}\;\mathrm{XNOR}\;b_{k},$$

which satisfies

(9) $$E[c_{k}]=ab.$$

Therefore,

(10) $$\hat{c}=\frac{1}{N}\sum_{k=1}^{N}c_{k}$$

is an unbiased estimator of ab.

#### 3.3.2. Addition via Stochastic Multiplexing

Weighted addition is implemented using probabilistic multiplexing:

(11) $$c_{k}=\left\{\begin{array}{cc} a_{k}, & \mathrm{with}\;\mathrm{probability}\;p,\\ b_{k}, & \mathrm{with}\;\mathrm{probability}\;1-p. \end{array}\right.$$

Then,

(12) $$E\left[c_{k}\right]=pa+\left(1-p\right)b.$$

#### 3.3.3. Integral Stochastic Computing

ISC introduces an integer-valued stochastic sample by aggregating *m* bipolar Bernoulli draws at each cycle:

(13) $$z_{k}^{\left(m\right)}=\sum_{r=1}^{m}s_{k,r},\;s_{k,r}\in \left\{-1,+1\right\},$$

with $z_{k}^{\left(m\right)}\in \left\{-m,-m+2,\ldots ,m\right\}$. The normalized estimator is

(14) $${\hat{x}}^{\left(ISC\right)}=\frac{1}{mN}\sum_{k=1}^{N}z_{k}^{\left(m\right)}.$$

Under independence,

(15) $$\mathrm{Var}\left[{\hat{x}}^{\left(ISC\right)}\right]=\frac{1-x^{2}}{mN},$$

so *m* provides a second precision knob in addition to *N*.

For arithmetic, ISC keeps stochastic multiplication at the sub-stream level and performs accumulation in the integer domain. Given two operands encoded as

(16) $$z_{a,k}^{\left(m\right)}=\sum_{r=1}^{m}a_{k,r},\;z_{b,k}^{\left(m\right)}=\sum_{r=1}^{m}b_{k,r},$$

with $a_{k,r},b_{k,r}\in \left\{-1,+1\right\}$, multiplication is formed by bitwise bipolar products (equivalently XNOR in {0,1} encoding):

(17) $$z_{ab,k}^{\left(m\right)}=\sum_{r=1}^{m}a_{k,r}b_{k,r},$$

and

(18) $$\hat{ab}=\frac{1}{mN}\sum_{k=1}^{N}z_{ab,k}^{\left(m\right)}.$$

Thus, multiplication remains stochastic at the bit level, while the output sample is integer-valued.

In contrast to SC MUX-based scaled addition, ISC addition is integer accumulation:

(19) $$z_{a+b,k}^{\left(m\right)}=z_{a,k}^{\left(m\right)}+z_{b,k}^{\left(m\right)},$$

so an *L*-term sum is accumulated directly as $\sum_{\ell =1}^{L}z_{\ell ,k}^{\left(m\right)}$ without probabilistic MUX scaling. Therefore, integer-domain accumulation preserves additive information that would otherwise be attenuated by repeated scaled additions in conventional SC.

## 4. Precision-Elastic Stochastic Quantum Simulation Framework

### 4.1. Design Space and Execution Axes

The proposed framework is organized around two key design dimensions that determine its computational behavior: (i) the arithmetic representation and (ii) the execution mode. These dimensions jointly define a configurable stochastic execution space with explicit control over the accuracy–cost trade-off.

The first dimension is the arithmetic family. Conventional stochastic computing provides a single precision control parameter through the bitstream length *N*, while integral stochastic computing introduces an additional aggregation parameter *m*, enabling two-dimensional precision control (N,m).

The second dimension is the execution mode for ISC, which determines how stochastic representations are propagated across gate operations. Two modes are considered: re-encoded (RE) and persistent (PS).

Combining these dimensions yields three primary execution modes summarized in Table 3: SC, ISC-RE, and ISC-PS.

RE and PS represent two fundamentally different execution strategies. RE restores approximate statistical independence between stages through decode/encode operations, while PS preserves stochastic representations across gates, enabling direct propagation without re-randomization. This distinction leads to different accuracy–cost characteristics and execution behaviors.

SC-PS is intentionally excluded. In SC, addition relies on MUX-based scaled operations, which introduce implicit attenuation. Without re-encoding, repeated operations cause exponential amplitude shrinkage, making persistent SC unsuitable for multi-layer quantum state propagation. Therefore, persistent execution is considered only within the ISC framework. Appendix A provides a concise schematic explanation of this attenuation mechanism.

In this work, ISC-PS serves as the primary framework of interest, while ISC-RE provides a reference execution mode for understanding the effect of stochastic dependence.

### 4.2. Accuracy–Cost Trade-Off via (N,m)

A key property of the proposed framework is explicit control of the accuracy–cost trade-off through the parameters (N,m).

In SC, estimation accuracy improves with increasing *N*, resulting in a variance scaling proportional to 1/N. In ISC, the additional aggregation parameter *m* further reduces variance, leading to an effective scaling proportional to 1/(mN) under ideal assumptions.

This two-dimensional control allows flexible adjustment of computational effort:

- Increasing *N* improves statistical accuracy through longer sampling.
- Increasing *m* improves representational resolution through integer aggregation.

Unlike fixed-point or floating-point representations, where precision is statically defined by bit width, the proposed framework enables continuous and runtime-adjustable precision. This property makes the framework suitable for scenarios where different levels of accuracy are required under varying computational budgets.

### 4.3. Stochastic Amplitude Encoding

Each complex amplitude $\psi_{i}=a_{i}+jb_{i}$ is encoded using stochastic representations for its real and imaginary components. A scalar value is represented as a length-*N* stochastic bitstream:

(20) $${\hat{a}}_{i}=\frac{1}{N}\sum_{k=1}^{N}s_{ik},$$

where $s_{ik}\in \left\{-1,+1\right\}$ and $E\left[{\hat{a}}_{i}\right]=a_{i}$.

The corresponding ISC representation extends this encoding by aggregating *m* stochastic samples per cycle, providing increased effective precision. Detailed arithmetic primitives and variance properties are described in Section 3.

### 4.4. Gate Application Models

Let $\psi_{k}$ denote the quantum state after the *k*-th gate and $U_{k}$ the corresponding unitary:

(21) $$\psi_{k+1}=U_{k}\psi_{k}.$$

#### 4.4.1. Re-Encoded Model (ISC-RE)

In RE execution,

(22) $${\hat{\psi }}_{k+1}=E\;\left(U_{k}\;D\left({\hat{\psi }}_{k}\right)\right),$$

where D and E denote decode and encode operators. This process restores approximate independence between stages and follows conventional stochastic sampling assumptions.

#### 4.4.2. Persistent Model (ISC-PS)

In persistent execution,

(23) $${\hat{\psi }}_{k+1}={\tilde{U}}_{k}\left({\hat{\psi }}_{k}\right),$$

where ${\tilde{U}}_{k}$ operates directly on stochastic representations without intermediate decode/encode steps.

**Figure 2:**
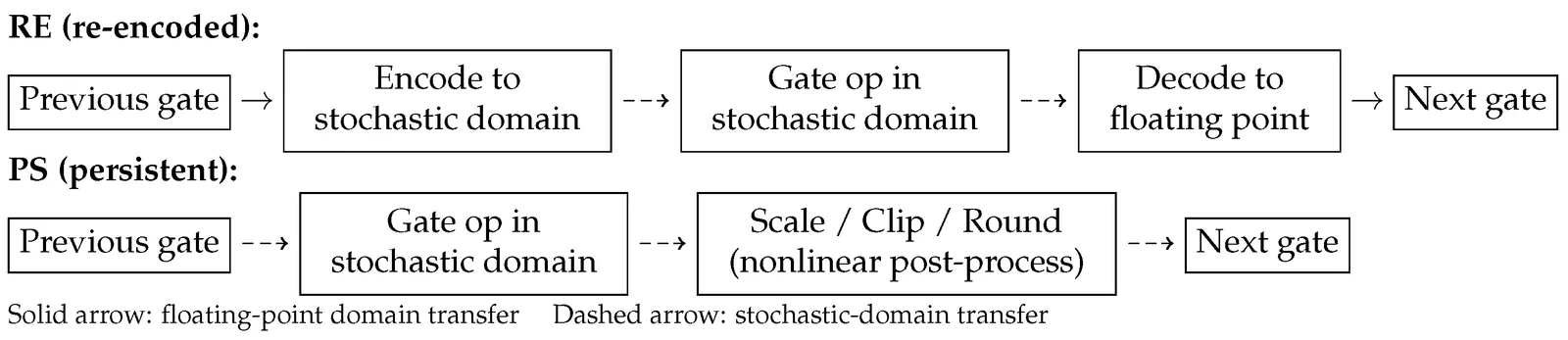
Gate-to-gate dataflow for RE and PS execution. RE transfers floating-point values between gates (solid arrows) with per-gate encode/compute/decode, while PS keeps stochastic-domain transfers (dashed arrows) so stream state persists across gates. In PS, an additional nonlinear post-processing step (scaling, clipping, and rounding) is applied within the stochastic domain, which contributes to cross-gate correlation and error amplification behavior.

This execution mode preserves stochastic dependence across layers and enables continuous propagation of bitstream states. As a result, ISC-PS exhibits distinct accuracy–cost characteristics compared to ISC-RE. Figure 2 summarizes the gate-to-gate distinction between re-encoded and persistent execution.

### 4.5. Persistent ISC: Practical Integer-Stream Realization

Persistent ISC incorporates practical hardware-inspired operations applied at each gate stage. Let $u_{k+1}$ denote the intermediate integer-domain accumulation. Persistent execution differs fundamentally from RE in that intermediate values are not decoded back to floating-point between gates. In RE execution, each gate output is rescaled and re-encoded, effectively resetting the dynamic range at every stage. In contrast, PS directly propagates integer-domain accumulations across layers. As a result, repeated additions and multi-term accumulations can cause the magnitude of intermediate values $u_{k}$ to grow beyond the representable stochastic range [−m,m]. Without additional control, this leads to saturation, loss of information, and unstable numerical behavior.

To maintain bounded representations and enable stable propagation, scaling and nonlinear post-processing (clipping and rounding) are required at each stage. These operations act as a practical normalization mechanism in persistent execution, but simultaneously introduce deterministic distortion and correlation-dependent effects. The post-processing is given by

(24) $${\tilde{u}}_{k+1}=\mathrm{round}\;\left(\mathrm{clip}\;\left(\frac{u_{k+1}}{\alpha_{k}},-m,m\right)\right),$$

where $\alpha_{k}$ is a scaling factor and clip(x,−m,m) truncates *x* to the interval [−m,m]. Additional operations such as deterministic renormalization and finite-bit quantization may be applied. With renormalization factor $g_{k}\geq 1$, the explicit decode scale is updated as

(25) $$S_{k+1}=\frac{S_{k}\;m}{g_{k}},$$

and decoded amplitudes are obtained by dividing stochastic-stream means by $S_{k}$.

These nonlinear operations introduce deviations from the ideal 1/(mN) variance model, and more importantly, create a mechanism for correlation-dependent error amplification across layers. As a result, the accuracy–cost behavior of ISC-PS depends not only on (N,m) but also on circuit structure and gate ordering.

### 4.6. Execution Scope

This work focuses on stochastic execution under SC, ISC-RE, and ISC-PS within a unified framework. The objective is not to replace deterministic simulation, but to establish a configurable stochastic simulation paradigm with controllable accuracy and computational cost.

Accordingly, all experiments analyze these execution modes under matched stochastic settings to evaluate their behavior within the proposed framework.

## 5. Accuracy–Cost Behavior and Design Analysis

This section analyzes the accuracy–cost behavior of the proposed stochastic computing framework, focusing on how parameters (N,m) and execution modes influence error characteristics. The analysis provides design-level insight into how the framework behaves under different operating conditions.

### 5.1. Bitstream Scaling Law

Let $e_{k}={\hat{\psi }}_{k}-\psi_{k}$ denote the stochastic error vector after *k* gates. For compact notation, define $E_{k}≜{∥e_{k}∥}_{2}^{2}$ as a theoretical simulation-side error metric relative to the exact statevector.

Under the re-encoded model and independent sampling assumptions, each gate introduces zero-mean stochastic perturbation with variance proportional to 1/N (or 1/(mN) for ISC).

Assuming approximate independence across gates, the expected squared error after *L* layers satisfies

(26) $$E{∥e_{L}∥}_{2}^{2}\approx \sum_{k=1}^{L}\sigma_{k}^{2},$$

where $\sigma_{k}^{2}\propto \frac{2^{n}}{N}$ for SC and

(27) $$\sigma_{k}^{2}\propto \frac{2^{n}}{mN}$$

for ISC.

Thus, under re-encoding,

(28) $$E{∥e_{L}∥}_{2}^{2}=O\;\left(\frac{L\;2^{n}}{N}\right)$$

for SC and

(29) $$E{∥e_{L}∥}_{2}^{2}=O\;\left(\frac{L\;2^{n}}{mN}\right)$$

for ISC.

This behavior provides a baseline accuracy–cost scaling law, where increasing *N* and *m* improves accuracy at increased computational cost.

### 5.2. Persistent Execution Behavior

Under persistent propagation, stochastic streams are reused across layers. Consequently, error terms become correlated:

(30) $$E\left[e_{i}^{\left(k\right)}e_{j}^{\left(k^{\prime }\right)}\right]\neq 0\;\mathrm{for}\;k\neq k^{\prime }.$$

The accumulated error is therefore not simply the sum of independent variances:

(31) $$E{∥e_{L}∥}_{2}^{2}=\sum_{k=1}^{L}\sigma_{k}^{2}+2\sum_{k<\ell }\mathrm{Cov}\left(e_{k},e_{\ell }\right).$$

This dependence introduces a distinct execution behavior compared to re-encoded operation. From a framework perspective, persistent execution provides an alternative operating mode with different accuracy–cost characteristics, influenced by circuit structure and stochastic dependence.

### 5.3. Quantization- and Clipping-Induced Effects in Persistent ISC

The ideal ISC model assumes variance scaling proportional to 1/(mN). However, persistent ISC includes additional nonlinear operations such as scaling, clipping, and rounding, which introduce deterministic bias.

Let *x* denote an intermediate quantity prior to post-processing. We define

(32) $$Q_{m}\left(x\right)≜\mathrm{round}\left(\mathrm{clip}\left(x,-m,m\right)\right),$$

and residual

(33) $$r_{m}\left(x\right)=Q_{m}\left(x\right)-x.$$

The evolution becomes

(34) $${\hat{\psi }}_{k+1}={\tilde{U}}_{k}\left({\hat{\psi }}_{k}\right)+\xi_{k}+r_{m,k},$$

where $\xi_{k}$ denotes stochastic perturbation noise arising from finite-length sampling. The total error can be decomposed into stochastic and quantization components as

(35) $$e_{k}=e_{k}^{\left(\mathrm{stoch}\right)}+e_{k}^{\left(\mathrm{quant}\right)},$$

Persistent execution accumulates $e_{k}^{\left(\mathrm{quant}\right)}$ across layers, while re-encoding suppresses it. The quantization error decreases with the integer precision *m* according to

(36) $$\mathrm{Var}\left[r_{m}\left(x\right)\right]\approx \frac{1}{12m^{2}}$$

Thus, the framework exhibits two distinct scaling terms:

(37) $$E{∥e_{L}∥}_{2}^{2}\approx C_{1}\frac{L\;2^{n}}{mN}+C_{2}\frac{L\;2^{n}}{m^{2}}+C_{3}\sum_{k<\ell }\mathrm{Cov}\left(e_{k},e_{\ell }\right)$$

where $C_{1}$, $C_{2}$, and $C_{3}$ are positive constants that collect gate- and circuit-dependent proportionality factors for the stochastic, quantization, and correlation terms, respectively. This decomposition highlights that accuracy is controlled not only by stochastic sampling (N,m) but also by execution mode and circuit-dependent correlation.

The required *m* depends on circuit-dependent intermediate magnitudes. This motivates adaptive parameter selection rather than fixed precision.

### 5.4. Circuit-Aware Parameter Selection

We provide a practical heuristic lower bound for selecting *m* in persistent ISC to avoid excessive clipping and rounding bias. Let $z_{k}$ denote the set of pre-post-processing intermediate accumulators after gate *k* (e.g., pre-clip complex matvec accumulators), and define the circuit-dependent peak as

(38) $$A_{\max}\;≜\;\max_{k}\;\max_{z\in z_{k}}\left|z\right|.$$

A sufficient condition to suppress clipping is

(39) $$m\;\geq \;\gamma \;A_{\max},$$

where γ>1 is a safety margin (e.g., γ∈[1.1,2]).

To constrain quantization-induced error under a target budget, we compare the $O(1/m^{2})$ term against $\varepsilon_{\mathrm{qc}}$, where $\varepsilon_{\mathrm{qc}}>0$ denotes a user-specified tolerance for the quantization/clipping error contribution:

(40) $$C_{2}\frac{L\;2^{n}}{m^{2}}\;\leq \;\varepsilon_{\mathrm{qc}}\;\Rightarrow \;m\;\geq \;\sqrt{\frac{C_{2}\;L\;2^{n}}{\varepsilon_{\mathrm{qc}}}}.$$

Combining both constraints gives

(41) $$m_{\min}\;≜\;\max\;\left\{\gamma \;A_{\max},\;\sqrt{\frac{C_{2}\;L\;2^{n}}{\varepsilon_{\mathrm{qc}}}}\right\}.$$

In practice, $A_{\max}$ can be estimated at low cost by a single floating-point scan that tracks pre-clip accumulator maxima across gates. This provides a conservative stabilization choice for *m* without exhaustive sweeps.

### 5.5. Model Deviation

Expectation-level models may deviate from actual stochastic execution:

(42) $$\Delta_{L}={∥{\bar{\psi }}_{L}-E\left[{\hat{\psi }}_{L}\right]∥}_{2}$$

where ${\bar{\psi }}_{L}$ denotes the state predicted by the simplified expectation-level analytical model at depth *L*, while ${\hat{\psi }}_{L}$ denotes the stochastic simulated state.

This motivates empirical evaluation of framework behavior.

### 5.6. Behavior Descriptors for Persistent Execution

To summarize persistent behavior, we use two empirical descriptors:

- ${\mathrm{slope}}_{\mathrm{tail}}$: scaling behavior with respect to *m*
- $\gamma_{c}$: circuit-dependent behavior descriptor capturing persistent sensitivity

Explicit definition of $\gamma_{c}$.

To make the extraction procedure explicit, we define $\gamma_{c}$ as an empirical tail coefficient computed over the high-*m* regime for persistent execution. Let ${\mathrm{err}}_{PS}\left(m\right)$ denote the mean squared error under persistent execution at a given *m*. Over a selected tail window $M_{\mathrm{tail}}$, we define

(43) $$\gamma_{c}={\mathrm{median}}_{m\in M_{\mathrm{tail}}}\left(m\;{\mathrm{err}}_{PS}\left(m\right)\right).$$

The tail window $M_{\mathrm{tail}}$ is selected after the transient crossover, where the scaling behavior becomes comparatively stable. In the present c1–c12 experiments, the tail window is fixed for all circuits as $M_{\mathrm{tail}}=$ {4096, 16,384, 65,536, 262,144}, corresponding to the evaluated high-*m* suffix used for descriptor extraction. This empirical definition captures the high-*m* persistent tail level in a compact form and is consistent with the tail-based characterization used throughout the experiments.

These descriptors provide a compact representation of how different circuits behave under the same framework settings, enabling comparative analysis within the proposed stochastic simulation framework.

## 6. Experimental Methodology

This section describes the evaluation methodology used to analyze the proposed stochastic computing framework, with particular focus on its accuracy–cost trade-off behavior and execution characteristics under different parameter settings.

### 6.1. Evaluation Metrics

We evaluate simulation behavior using the following metrics:

- Squared Euclidean error ${∥\hat{\psi }-\psi ∥}_{2}^{2}$,
- Fidelity $F(\hat{\psi },\psi )$,
- Trace distance,
- Runtime per circuit execution.

For normalized pure-state comparisons, fidelity is defined as

(44) $$F\left(\hat{\psi },\psi \right)={\left|\langle \psi \;|\;\hat{\psi }\rangle \right|}^{2},$$

and the corresponding trace distance is

(45) $$D\left(\hat{\psi },\psi \right)=\sqrt{1-F(\hat{\psi },\psi )}.$$

For stochastic execution modes, all reported values are mean ± standard deviation over independent seeds.

### 6.2. Workloads

We use both controlled synthetic workloads and benchmark circuits from QASMBench [7]. Synthetic circuits (e.g., n=4, L=4) are used for controlled scaling validation, while QASMBench circuits provide realistic gate composition and depth variation.

The evaluation spans c1–c12 circuits, as listed in Table 4. This range is sufficient to capture circuit-dependent behavior, as the observed accuracy–cost characteristics are primarily influenced by circuit structure and interaction patterns rather than state dimension alone.

### 6.3. Precision Parameters

The framework exposes two precision-control parameters:

- Bitstream length *N*,
- ISC aggregation factor *m*.

These parameters jointly determine the accuracy–cost trade-off of stochastic execution. Scaling experiments sweep *N* and *m* to evaluate their impact on simulation behavior.

### 6.4. Multi-Seed and Reproducibility Protocol

Persistent experiments use S=10 independent random seeds. For each (circuit,N,m) configuration, results are averaged across seeds and reported with standard deviation.

All simulations were performed on a macOS 26.3.1 system with an Apple M2 Max processor (Apple Inc., Cupertino, CA, USA) (12 CPU cores) and 96 GB memory. The software environment includes Python 3.10.7 with NumPy 2.2.4. All experiments in this work are conducted using our simulator, *StoqSim* (Stochastic Quantum Simulator), which supports both re-encoded and persistent stochastic execution modes. The source code is available at https://github.com/nonizawa/persistent-isc-quantum-sim (accessed on 25 February 2026) [24].

### 6.5. m-Sweep Protocol

At fixed *N*, we sweep *m* on a logarithmic grid to evaluate accuracy scaling and persistent execution behavior.

The high-*m* region is used to extract summary descriptors such as ${\mathrm{slope}}_{\mathrm{tail}}$ and $\gamma_{c}$, which characterize circuit-dependent behavior under consistent stochastic settings.

### 6.6. Experimental Protocol for Target-Fidelity Adaptive Precision Scheduling

To evaluate precision elasticity as an operational scheduling mechanism, we run a target-fidelity adaptive precision scheduling experiment on c1–c12 for both ISC-RE and ISC-PS. The common target is fixed as $F_{\mathrm{target}}=0.9995$, selected to represent a high-fidelity operating regime while still exposing observable precision-selection differences across circuits. For each circuit and mode, we search the two-parameter precision grid

$$\begin{array}{cc} N & \in \{1000,2000,5000,10,000,20,000\},\\ m & \in \{64,128,256,512,1024,2048\}, \end{array}$$

and select

$$\left(N^{*},m^{*}\right)=\underset{N,m}{\arg\;\min}\;Nm\;s.t.\;F\left(N,m\right)\geq F_{\mathrm{target}}.$$

As the fixed high-precision reference, we use $(N_{\mathrm{fixed}},m_{\mathrm{fixed}})=$ (10,000, 1024). The stochastic cost proxy is C=Nm, and the cost reduction ratio is

$$R_{\mathrm{cost}}=\frac{N_{\mathrm{fixed}}m_{\mathrm{fixed}}}{N*m*}.$$

Although C=Nm does not directly represent physical runtime or energy, it provides a first-order stochastic workload proxy because *N* determines temporal sampling length while *m* determines per-cycle integer accumulation complexity. The objective is not deterministic numerical equivalence to FP16, but resource-aware stochastic precision selection under a user-defined fidelity requirement. Because this experiment uses the same stochastic evaluation protocol as the main benchmark with a single seed/repeat setting, the selected operating points should be interpreted as empirical scheduling choices under the evaluated seed/repeat condition.

### 6.7. Evaluation Axes

Experiments are organized along four complementary evaluation axes:

1. Arithmetic comparison: Comparison between SC, ISC-RE, and ISC-PS under matched stochastic settings.
2. Accuracy–cost behavior: Analysis of how parameters (N,m) affect simulation accuracy and runtime.
3. Execution-mode characterization: Comparison between ISC-RE and ISC-PS to understand the impact of persistent stochastic propagation.
4. Consistency with deterministic baselines: Comparison with FP16 and the fixed-point baseline (16 fractional bits) as trend-consistency references. FP16 and the fixed-point baseline (16 fractional bits) are implemented in-house within the same simulation pipeline. FP16 uses NumPy-based float16 emulation, while the fixed-point baseline uses uniform quantization with a fixed number of fractional bits.

These axes provide a structured evaluation of the proposed framework, linking theoretical analysis (Section 5) with empirical observations.

## 7. Results

### 7.1. Consistency with Fixed-Point and Floating-Point Representations

We use FP16 and a fixed-point baseline only as reduced-precision trend-consistency references. The goal is not to demonstrate deterministic numerical equivalence, but to check whether the stochastic execution modes retain meaningful cross-circuit tendencies at the common operating point (N,m)= (10,000, 1024). Accordingly, agreement is summarized by Pearson and Spearman correlation across c1–c12.

Table 5 summarizes the resulting correlations with FP16. The deterministic fixed-point baseline remains closely aligned with FP16, whereas ISC-RE and ISC-PS show weaker but nonzero trend consistency. At the common operating point, ISC-PS gives a slightly higher Pearson correlation than ISC-RE, while the Spearman values are comparable. These observations are used only as a secondary consistency check; the main results of this work concern persistent stochastic execution dynamics and correlation-sensitive propagation rather than FP16 matching.

**Figure 3:**
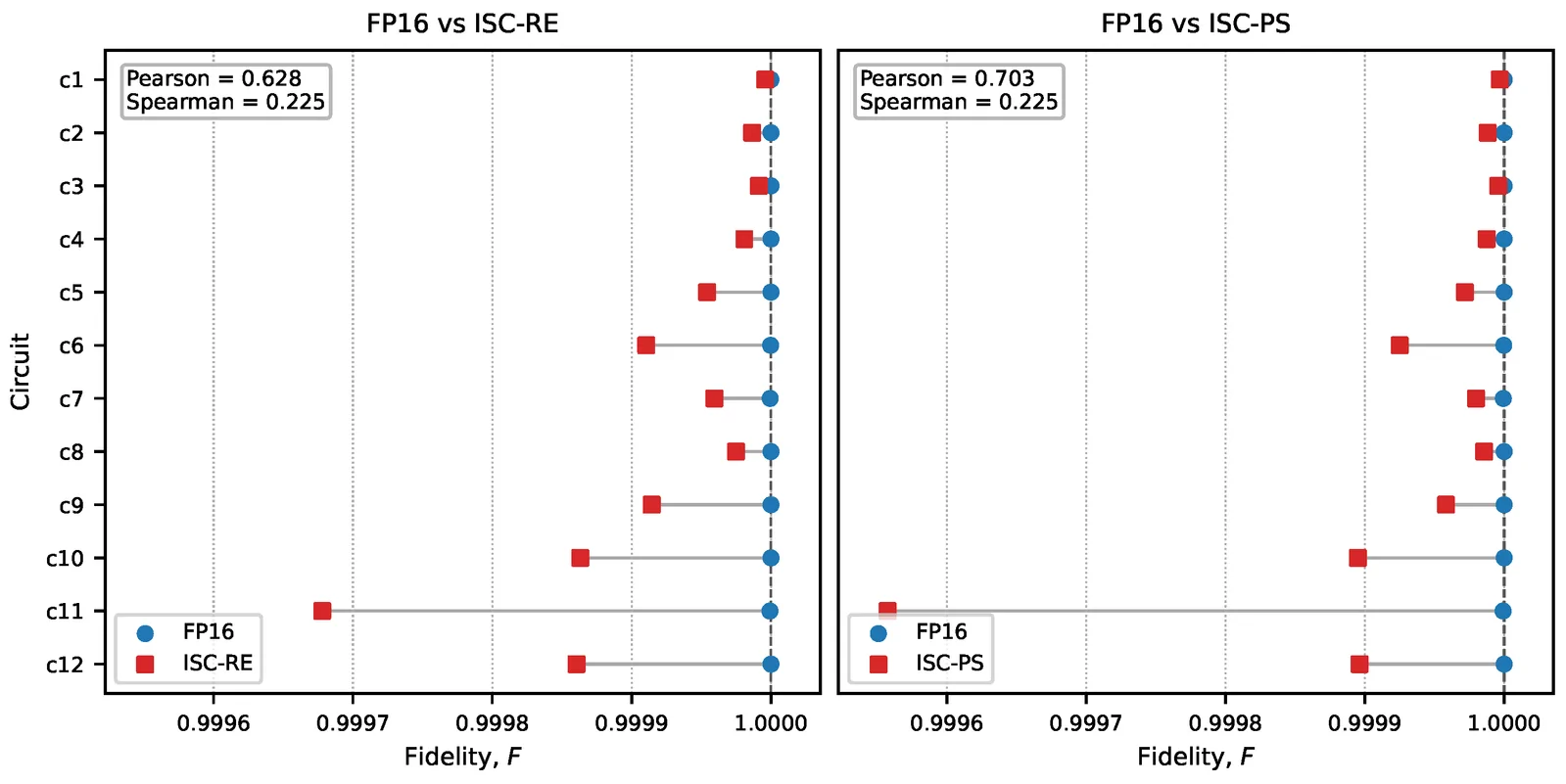
FP16 trend-consistency reference for ISC-RE and ISC-PS at the common operating point (N,m)= (10,000, 1024). The comparison is used only as a secondary consistency reference and is not intended to demonstrate deterministic numerical equivalence.

Figure 3 shows the corresponding circuit-wise fidelity comparison for ISC-RE and ISC-PS. The figure provides a compact visual reference for the same point: stochastic execution preserves partial cross-circuit trend information under matched controls, without being interpreted as a deterministic reduced-precision substitute.

### 7.2. SC and ISC Comparison on Representative Circuits

Before analyzing persistent behavior, we first compare the arithmetic behavior of standard stochastic computing (SC) [25] and re-encoded integral stochastic computing (ISC-RE) on representative workloads. The detailed examples in this subsection use c1 and c4 to contrast a compact shallow circuit with a more correlation-sensitive case, while the cross-circuit persistent analysis in the following subsections uses the full c1–c12 benchmark set. SC encodes each amplitude component into a single bipolar bitstream, so precision is controlled only by stream length. ISC-RE adds an integer accumulation axis through *m* and restores approximate inter-gate independence through re-encoding, which increases effective representational granularity under the same stochastic execution setting.

**Figure 4:**
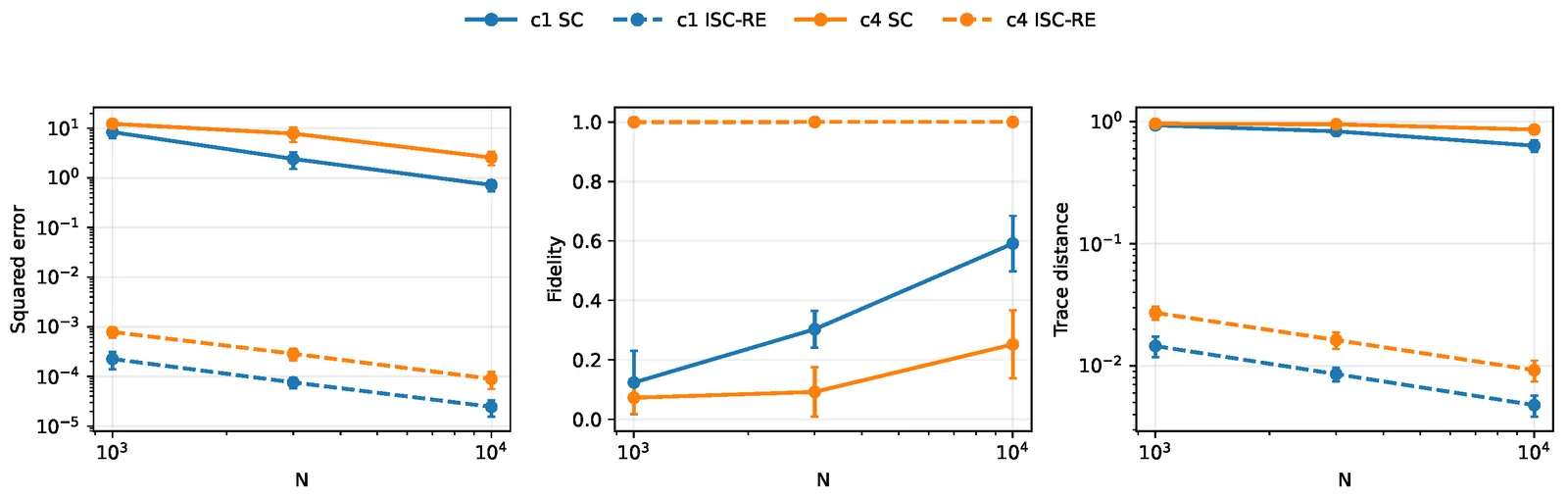
Representative SC vs. ISC-RE comparison (c1 and c4) across stochastic precision settings. (**left**,**middle**,**right**) panels report squared error, fidelity, and trace distance, respectively. Colors distinguish circuits (c1, c4), while line styles distinguish execution modes (SC, ISC-RE). ISC-RE shows substantially improved state accuracy while preserving consistent fidelity/trace trends.

Under matched stochastic precision settings, ISC-RE typically reduces numerical error relative to SC because integer-domain accumulation mitigates the scaling loss that appears in repeated stochastic operations. This comparison is used here as a baseline comparison to establish arithmetic characteristics: the goal is to clarify arithmetic behavior differences, not to claim replacement of deterministic simulation. Figure 4 summarizes this representative SC–ISC-RE comparison.

To keep the quantum-state interpretation complete, this comparison should be read using squared error together with fidelity and trace distance, since those metrics capture complementary aspects of state deviation.

At N= 10,000, the representative runs show large SC–ISC-RE separation: for c1, squared error decreases from $7.19\times 10^{-1}$ (SC) to $2.45\times 10^{-5}$ (ISC-RE), and for c4, from 2.56 (SC) to $8.95\times 10^{-5}$ (ISC-RE). The corresponding fidelities move from 0.591/0.252 (SC, c1/c4) to 0.99998/0.99991 (ISC-RE), with matching trace-distance reduction.

These observations motivate the next step: ISC execution itself has distinct regimes, re-encoded and persistent, which are analyzed next.

### 7.3. Persistent ISC as a Distinct Execution Regime

**Figure 5:**
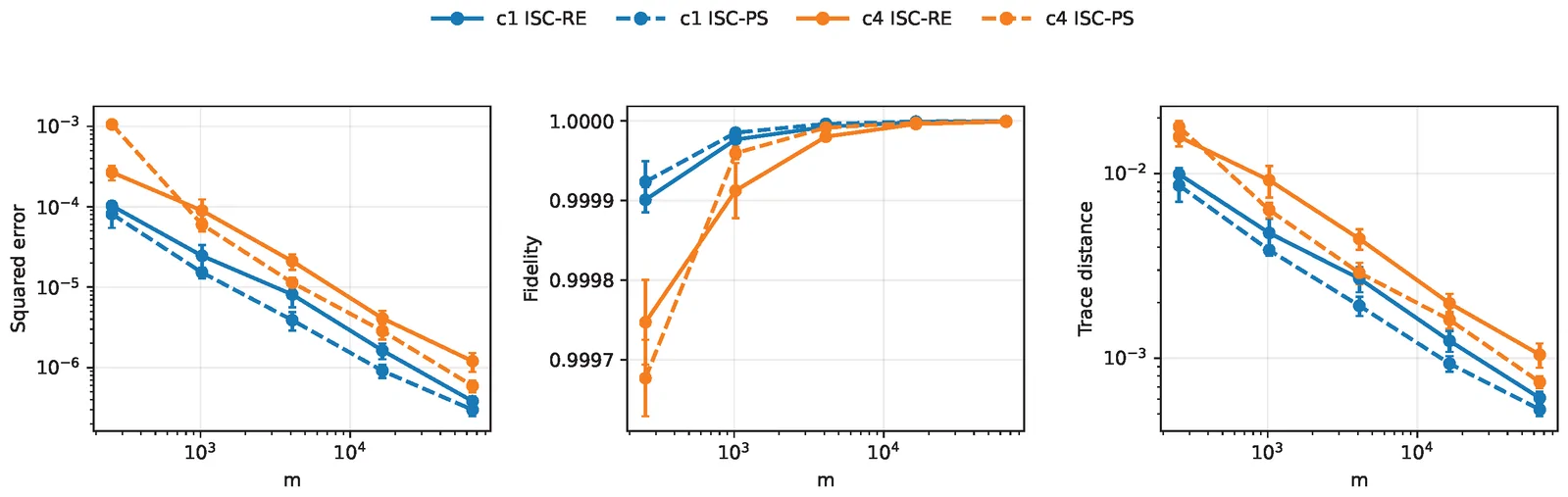
Representative ISC-RE vs. ISC-PS comparison (c1 and c4) over *m* at fixed *N* = 10,000. Squared error, fidelity, and trace distance are shown together to highlight regime-specific behavior beyond a single error metric. Colors distinguish circuits (c1, c4), while line styles distinguish execution regimes (ISC-RE, ISC-PS).

This subsection compares two ISC execution regimes under matched stochastic settings: ISC-RE and ISC-PS. In ISC-RE, re-encoding between stages re-randomizes streams and approximately restores stage-wise independence, which suppresses accumulation of cross-stage correlation. In ISC-PS, stochastic streams are propagated without this reset, so correlation terms can persist and accumulate across gate stages. Figure 5 provides representative regime-level trajectories for c1 and c4.

At fixed *N*, let ${\mathrm{err}}_{PS}\left(m\right)$ and ${\mathrm{err}}_{RE}\left(m\right)$ denote nonnegative squared-error metrics. The primary characterization of persistent behavior is given by the tail descriptors $\left({\mathrm{slope}}_{tail},\gamma_{c}\right)$ extracted from the high-*m* region.

For intuitive comparison at finite *m*, we also consider the relative ratio

(46) $$\mathrm{ratio}\left(m\right)=\frac{{\mathrm{err}}_{PS}\left(m\right)}{{\mathrm{err}}_{RE}\left(m\right)},$$

which provides a local, point-wise comparison between persistent and re-encoded execution at each operating point. Because errors are nonnegative by definition, this ratio is mathematically well defined without extra absolute-value notation.

**Figure 6:**
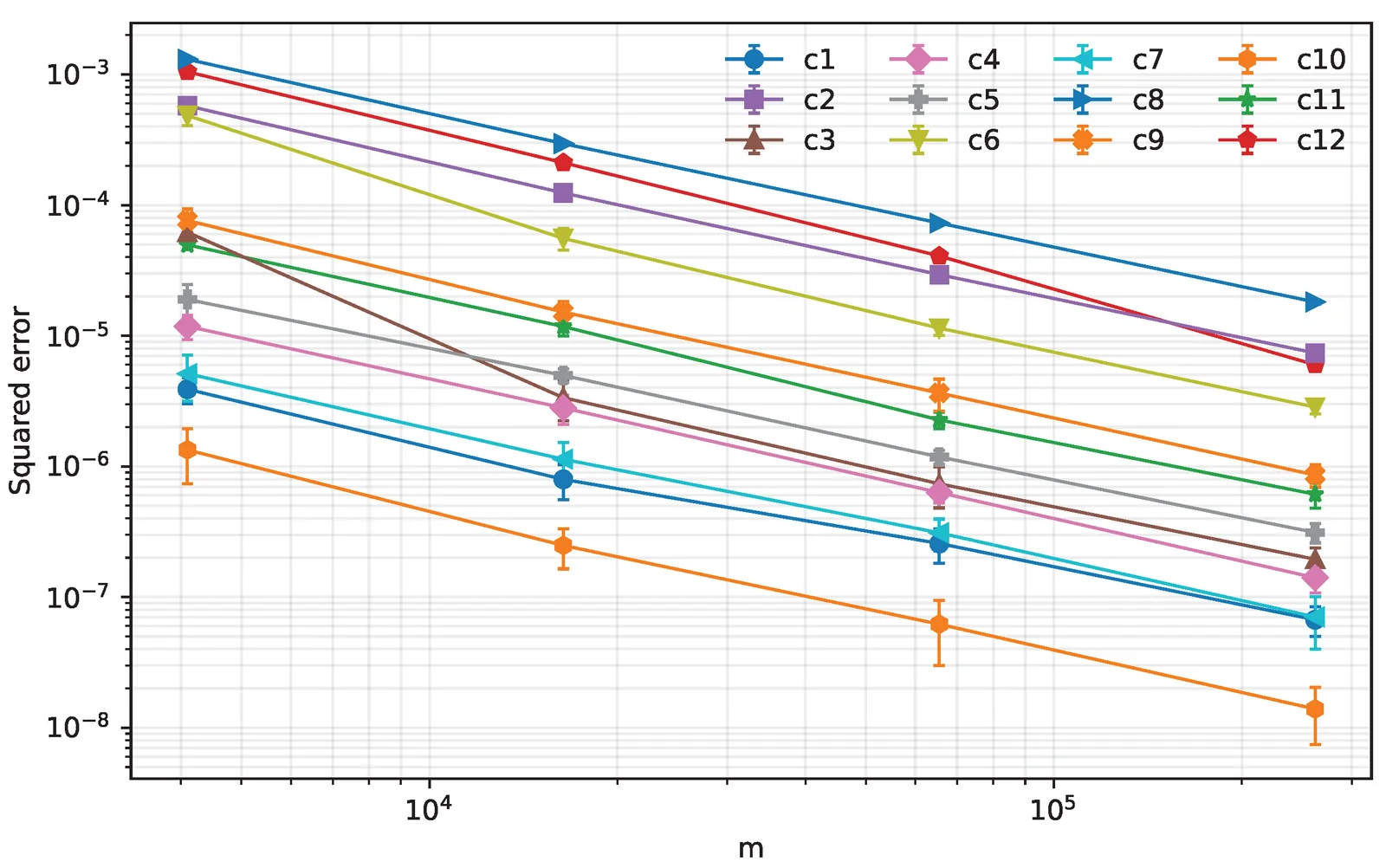
Persistent ISC *m*-sweep across c1–c12 at fixed *N*. The curves show circuit-dependent tail regimes (convergent, plateau-like, and crossover), which are summarized by ${\mathrm{slope}}_{tail}$ and $\gamma_{c}$ in the following analysis.

Figure 6 shows the persistent *m*-sweep across c1–c12. The observed behaviors (convergent, plateau-like, and crossover) indicate that ISC-PS is not merely a degraded version of ISC-RE, but a qualitatively different stochastic execution regime with its own propagation characteristics.

Persistent execution can therefore amplify correlation-sensitive circuit structures: when interaction patterns and depth promote correlation carryover, the PS trajectory can deviate systematically from the RE reference. This dependence on circuit structure is a core empirical property of the persistent regime.

In these representative sweeps, ISC-PS does not behave as a uniform degradation of ISC-RE. For c1, the ${\mathrm{err}}_{PS}/{\mathrm{err}}_{RE}$ ratio remains below unity across the scanned *m* range. For c4, the ratio varies non-monotonically and crosses both above and below unity, indicating regime-specific dependence on circuit structure and precision setting.

The next analysis quantifies how this regime behavior varies across circuits through tail scaling summaries, leading to the $\gamma_{c}$-based characterization.

### 7.4. Quantifying Persistence-Related Behavior: $\gamma_{c}$ and Tail Slope

**Figure 7:**
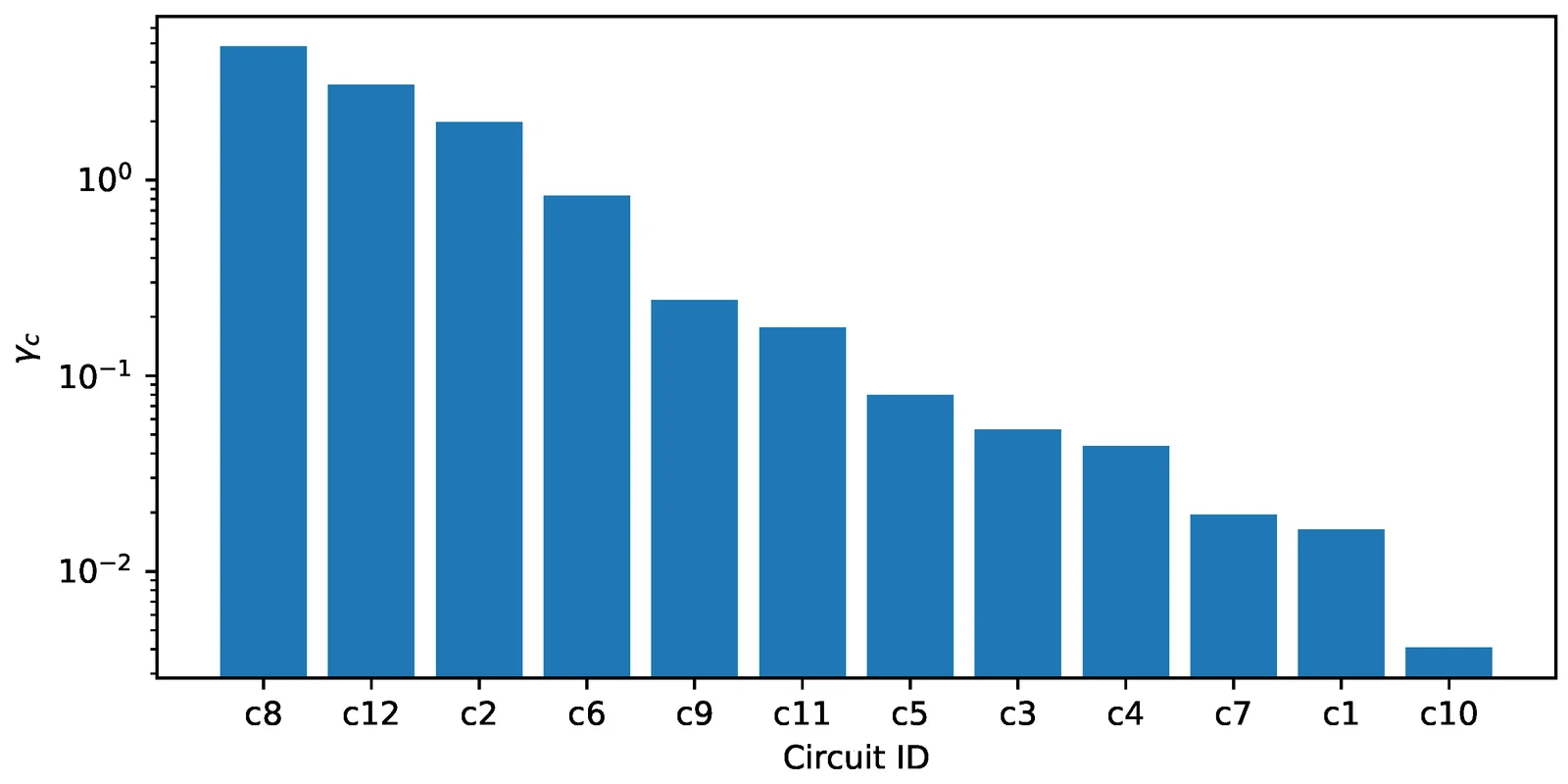
Circuit-wise persistent tail coefficient $\gamma_{c}$ for c1–c12 (ordered as c1, c2, …, c12). Higher values indicate a larger persistent high-*m* tail level under matched settings, enabling a compact cross-circuit robustness comparison. Here, $\gamma_{c}$ is computed using the fixed tail window $M_{\mathrm{tail}}=$ {4096, 16,384, 65,536, 262,144} for all circuits.

To summarize persistence strength per circuit, we use $\gamma_{c}$ together with ${\mathrm{slope}}_{tail}$. Figure 7 ranks circuits by $\gamma_{c}$. Larger $\gamma_{c}$ indicates a higher persistent tail level under matched controls, while smaller $\gamma_{c}$ indicates relatively robust behavior. These values are computed from the same multi-seed sweep used in Figure 6. Structurally, $\gamma_{c}$ summarizes the magnitude of the persistent high-*m* tail and therefore serves as a compact descriptor of circuit-dependent tail behavior under repeated stochastic reuse.

To make full-set coverage explicit, Table 6 reports a fixed operating-point snapshot (N= 10,000, *m* = 1024) for all 12 circuits in this study.

In addition, Table 6 highlights the substantial accuracy gap between conventional SC and ISC-based execution. Across all circuits, both ISC-RE and ISC-PS reduce squared error by several orders of magnitude compared to SC under the same operating point. This confirms that integer-domain accumulation in ISC effectively mitigates the scaling loss inherent in SC arithmetic, providing significantly improved numerical stability.

Interestingly, the relative performance ordering between ISC-RE and ISC-PS was not uniform across circuits. In this snapshot, four circuits satisfy ${\mathrm{err}}_{PS}>{\mathrm{err}}_{RE}$, while the median ${\mathrm{err}}_{PS}/{\mathrm{err}}_{RE}$ ratio across the 12-circuit set is 0.607, reinforcing that persistent behavior is circuit-dependent rather than uniformly shifted.

The additional ${\mathrm{err}}_{PS}/{\mathrm{err}}_{SC}$ column makes the same snapshot comparable to SC, while runtime columns provide insight into computational cost differences across execution modes. In particular, ISC-PS achieves substantially lower runtime than ISC-RE because it eliminates per-gate encode/decode operations, allowing direct propagation of stochastic representations across layers. At the same time, the runtime of ISC-PS remains comparable to that of SC, indicating that persistent execution introduces only modest overhead relative to conventional stochastic computation while providing significantly improved accuracy.

**Figure 8:**
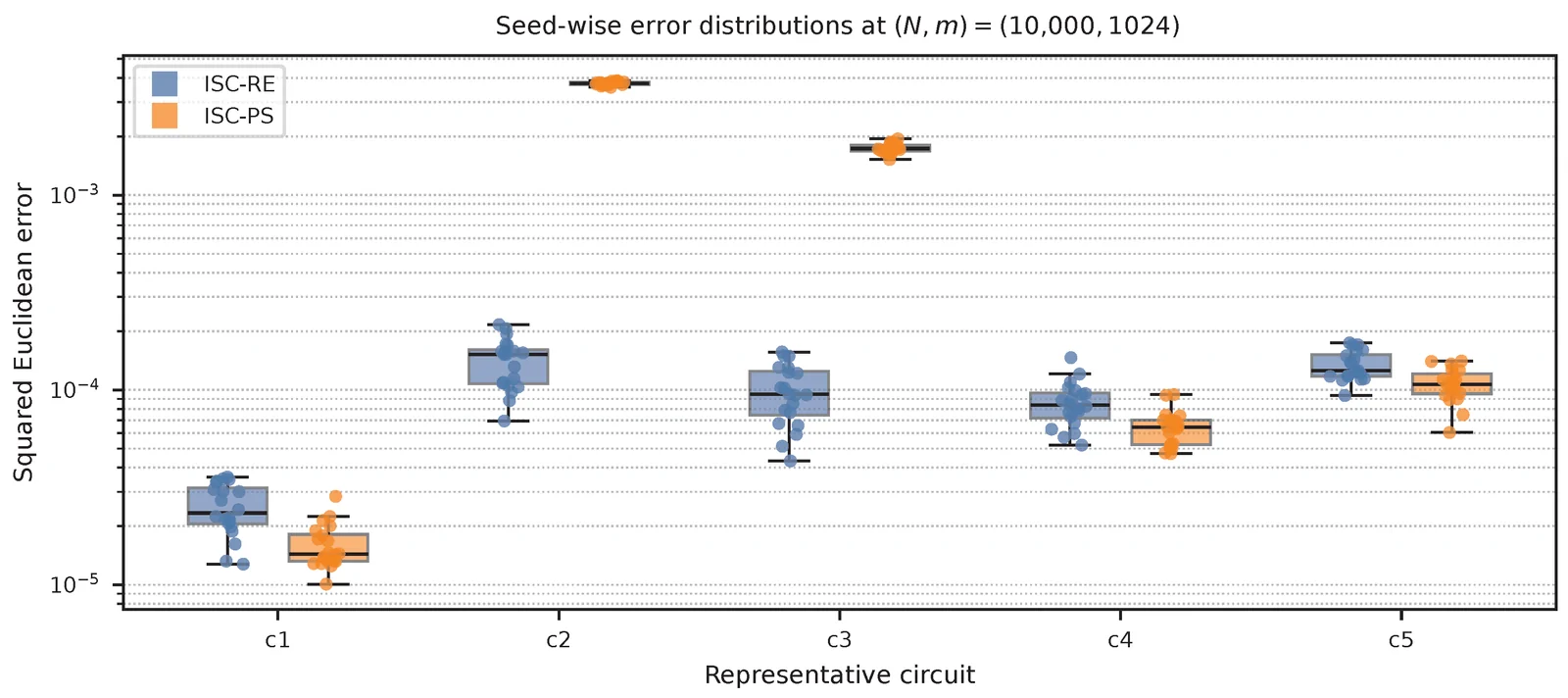
Seed-wise squared-error distributions for ISC-RE and ISC-PS at (N,m)= (10,000, 1024). The box-plots use representative circuits c1–c5 with 20 seeds per execution mode. The circles denote outlier seed values outside the whiskers, where the whiskers follow the standard 1.5 interquartile-range rule. The distributions provide error-spread information complementary to the mean values in Table 6.

To complement the mean-error snapshot in Table 6, we also examine seed-wise error distributions at the same fixed operating point, (N,m)= (10,000, 1024). Figure 8 reports box-plots of squared Euclidean error for representative circuits c1–c5 using 20 seeds per mode. The distributions show that persistent execution changes not only the mean error but also the spread and location of the seed-wise error samples in a circuit-dependent manner. For c2 and c3, ISC-PS is shifted to a substantially higher error range than ISC-RE, whereas for c1, c4, and c5 the ISC-PS distribution has a lower median and comparable or smaller interquartile spread. Thus, the persistent regime is not characterized by a uniform variance increase; instead, its distributional effect depends on circuit structure and accumulated cross-layer correlation.

To further interpret $\gamma_{c}$, we examine a tail-window dispersion metric derived from seed-to-seed variability in the persistent regime. This metric is computed using the same high-*m* tail window used for the $\gamma_{c}$ estimation. More concretely, let $\sigma_{e}\left(m\right)$ denote the seed-wise standard deviation of squared error at a fixed *m* within the high-*m* tail window. The dispersion metric is defined as

(47) $$D_{c}={\mathrm{median}}_{m\in \mathrm{tail}}\left(m\;\sigma_{e}\left(m\right)\right),$$

and the plotted values correspond to $\log_{10}\left(D_{c}\right)$.

**Figure 9:**
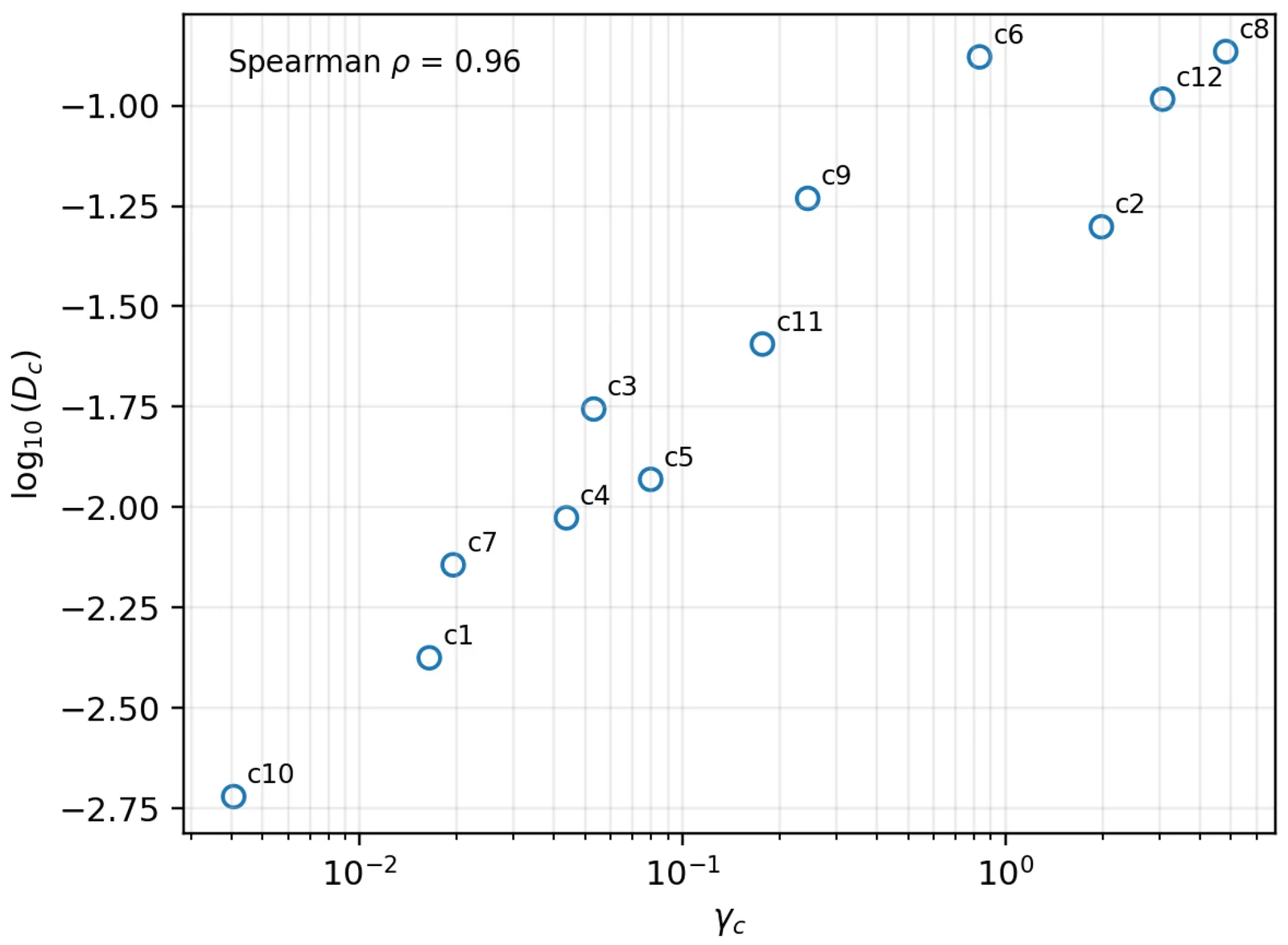
$\gamma_{c}$ versus the tail-window dispersion metric $D_{c}$ computed from persistent execution across c1–c12. The vertical axis shows $\log_{10}\left(D_{c}\right)$, where $D_{c}$ is defined as the median over the persistent tail window of $m\;\sigma_{e}\left(m\right)$, with $\sigma_{e}\left(m\right)$ denoting the seed-wise standard deviation of squared error at fixed *m*. The same fixed tail window $M_{\mathrm{tail}}=$ {4096, 16,384, 65,536, 262,144} is used for all circuits. This dispersion reflects the sensitivity of persistent execution to accumulated cross-layer dependence. The monotonic trend indicates that larger $\gamma_{c}$ values align with stronger tail variability under persistent execution.

Figure 9 provides an important consistency check using the same persistent tail window as the $\gamma_{c}$ fit. The dispersion metric captures seed-to-seed variability induced by accumulated cross-layer dependence under persistent execution through the tail-window aggregate of $m\;\sigma_{e}\left(m\right)$. The observed monotonic association indicates that larger $\gamma_{c}$ values correspond to stronger tail variability, supporting the interpretation of $\gamma_{c}$ as a descriptor of correlation-sensitive behavior rather than only a curve-fit coefficient. This association suggests that persistent stochastic execution induces reproducible circuit-dependent variability regimes, which can be compactly summarized through $\gamma_{c}$. Thus, $\gamma_{c}$ should be interpreted as a descriptor of persistent stochastic information propagation under repeated reuse, not as a direct predictor of mean error increase.

The relationship between Figure 9 and the cross-circuit summaries in Table 6 (and related comparisons) highlights a key distinction between variability and mean error amplification. The coefficient $\gamma_{c}$ and the dispersion metric in Figure 9 capture the sensitivity of persistent execution to accumulated cross-layer dependence, reflected as increased seed-to-seed variability in the high-*m* tail regime. In contrast, the ratio err*_PS_*/err*_RE_* reported in Table 6 reflects the realized mean error difference between persistent and re-encoded execution. While circuits with larger $\gamma_{c}$ tend to exhibit stronger variability and correlation sensitivity, this does not necessarily translate into a proportionally larger mean error ratio, since the latter also depends on circuit-specific cancellation effects and interaction structure. Therefore, $\gamma_{c}$ should be interpreted as a tail-level sensitivity descriptor rather than a direct predictor of mean error increase. Accordingly, larger $\gamma_{c}$ values indicate stronger circuit-dependent sensitivity to persistent stochastic propagation, rather than simply poorer average execution accuracy.

### 7.5. Target-Fidelity Adaptive Precision Scheduling

We next evaluate target-fidelity adaptive precision scheduling using a common fidelity requirement $F_{\mathrm{target}}=0.9995$ for all c1–c12 circuits and for both ISC-RE and ISC-PS. For each circuit and mode, the scheduler searches the predefined (N,m) candidate grid and selects the minimum stochastic cost proxy C=Nm subject to $F\geq F_{\mathrm{target}}$, then compares this selected operating point to the fixed high-precision setting (N,m)= (10,000, 1024).

**Figure 10:**
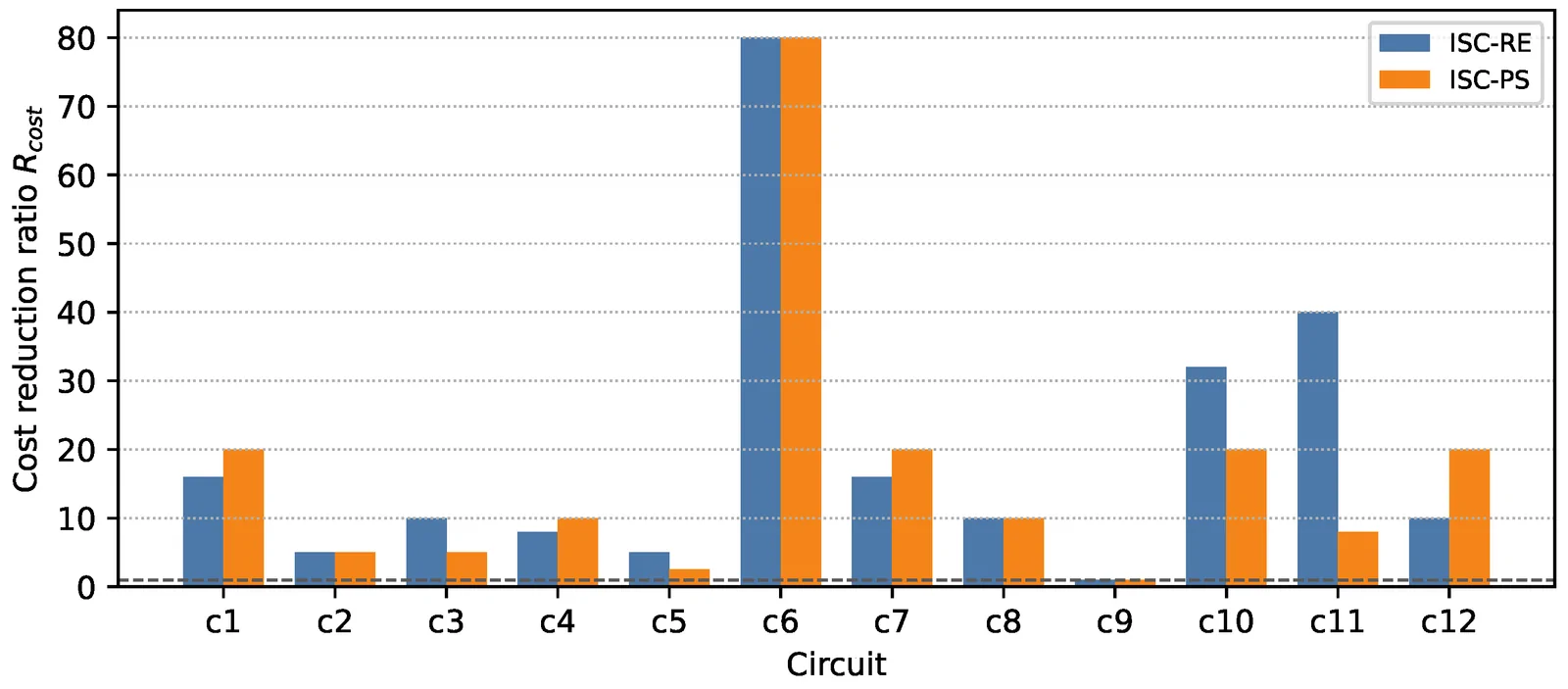
Target-fidelity adaptive precision scheduling for c1–c12. For each circuit and execution mode, the lowest-cost (N,m) pair satisfying F≥0.9995 is selected from the candidate grid. The cost reduction ratio $R_{\mathrm{cost}}$ is computed relative to the fixed high-precision setting (N,m)= (10,000, 1024), using the stochastic cost proxy C=Nm rather than measured wall-clock runtime. The dashed horizontal line marks $R_{\mathrm{cost}}=1$, corresponding to the reference cost of the fixed high-precision setting. Values above one indicate reduced stochastic cost while satisfying the target fidelity.

**Figure 11:**
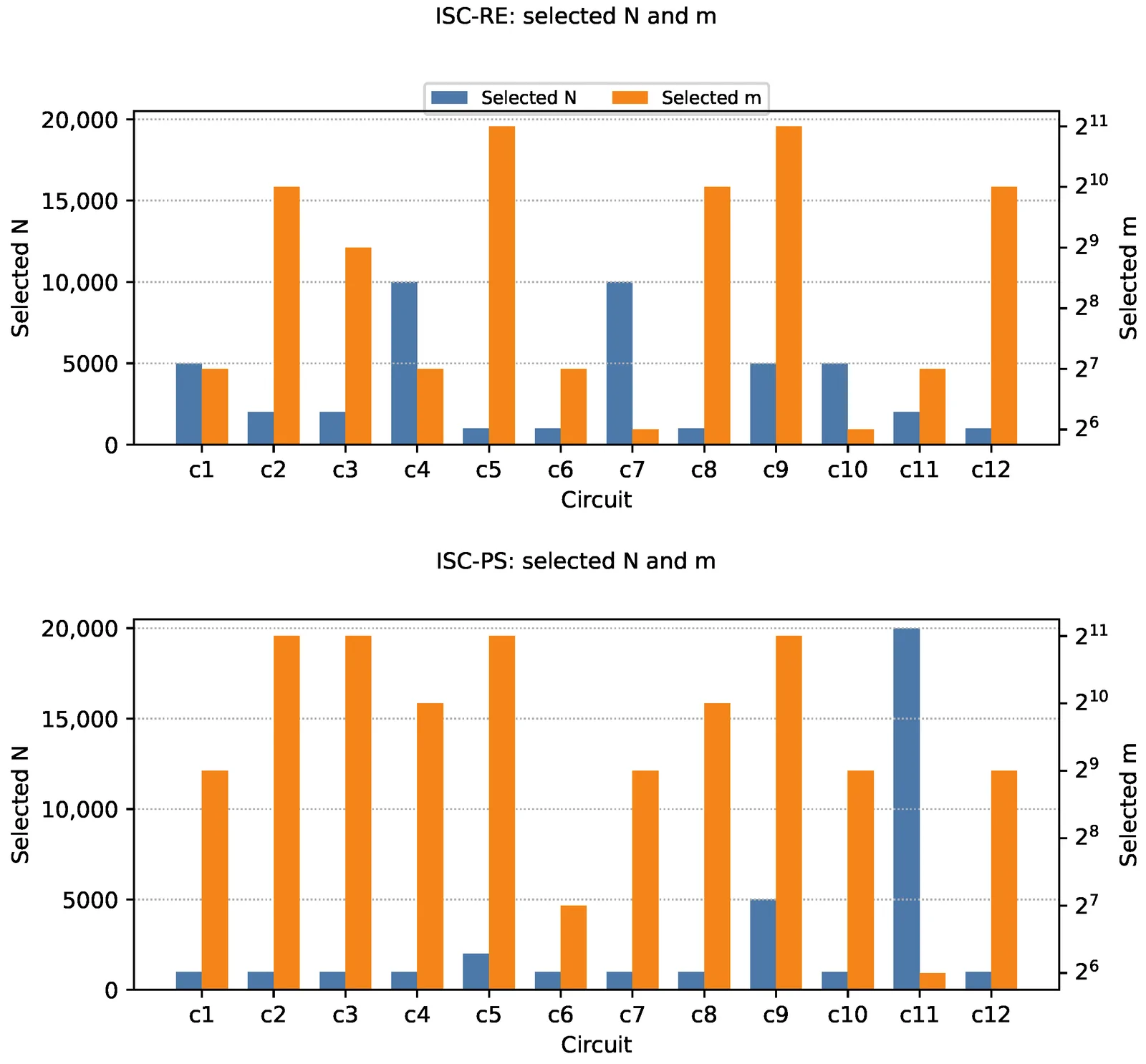
Selected circuit-dependent (N,m) pairs under target-fidelity adaptive precision scheduling. Top: ISC-RE. Bottom: ISC-PS. Different circuits select different operating points even under the same $F_{\mathrm{target}}=0.9995$, indicating workload-dependent stochastic precision requirements. Unlike Table 7, this figure reports minimum-cost operating points rather than minimum feasible *m* alone.

All selected operating points satisfy the target fidelity constraint in this experiment. The selected (N,m) pairs are clearly circuit-dependent, which confirms that stochastic precision demand is workload-dependent even under a common target-fidelity scheduling condition. Across c1–c12, the mean (median) cost reduction ratio $R_{\mathrm{cost}}$ is 19.42 (10.0) for ISC-RE and 16.79 (10.0) for ISC-PS. Figure 10 summarizes the circuit-wise cost reduction ratios, and Figure 11 shows the selected (N,m) pairs.

**Figure 12:**
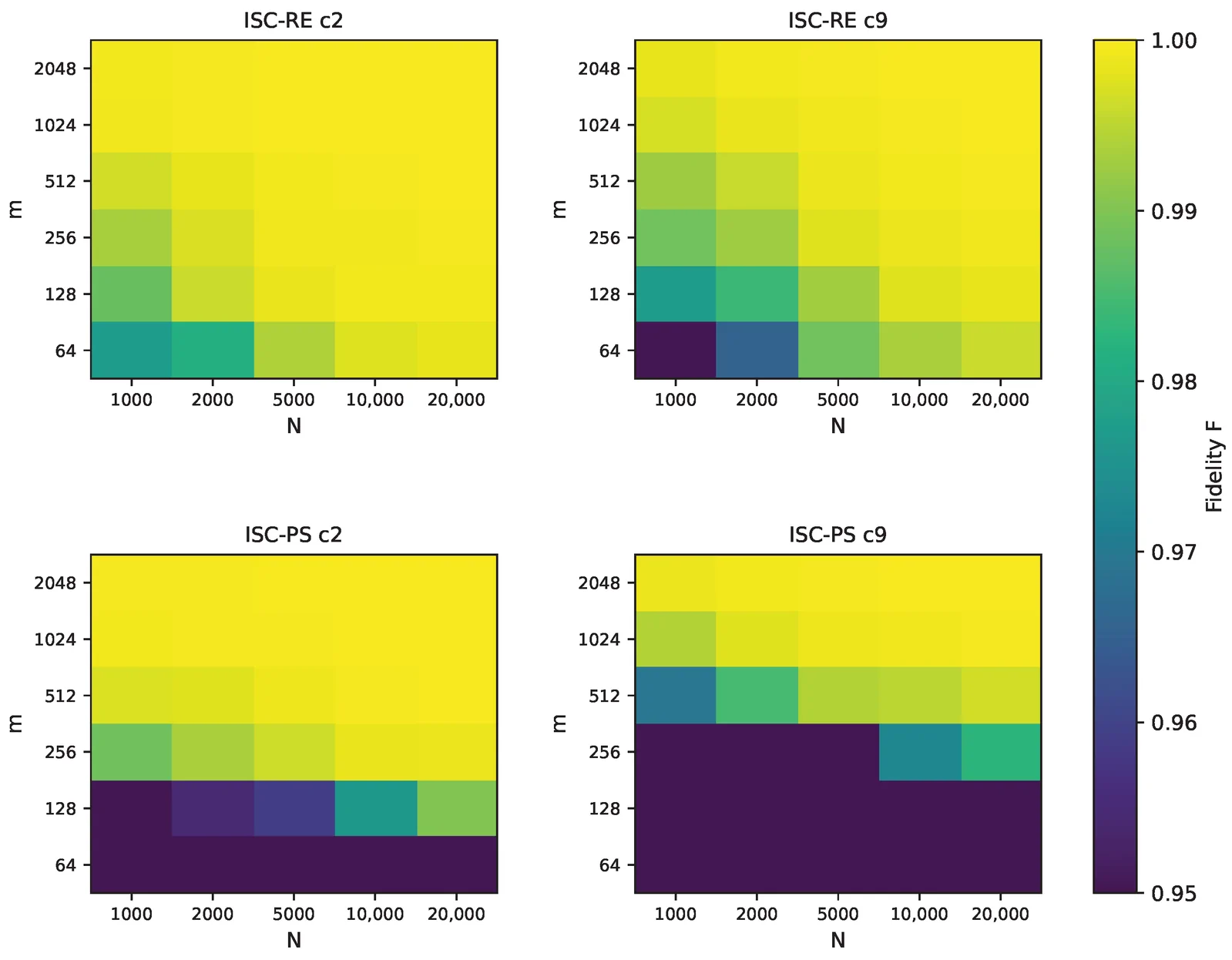
Representative fidelity heatmaps over the (N,m) grid for c2 and c9 under ISC-RE and ISC-PS. The maps visualize how feasibility with respect to $F_{\mathrm{target}}=0.9995$ depends on both parameters, and why circuit-dependent (N,m) selection is required.

Qualitatively, ISC-RE shows smoother feasibility progression over the (N,m) grid, while ISC-PS requires larger *m* on several circuits, consistent with stronger sensitivity to low-*m* persistent stochastic propagation. Representative heatmaps in Figure 12 illustrate this grid-level behavior for c2 and c9 in both modes. This scheduling analysis is presented from two complementary perspectives. Table 7 isolates the empirical minimum feasible multiplicity $m_{\min}\left(c;F_{\mathrm{target}}\right)$, defined as $m_{\min}\left(c;F_{\mathrm{target}}\right)=\min\left\{m:\exists N\;\mathrm{such}\;\mathrm{that}\;F\left(N,m\right)\geq F_{\mathrm{target}}\right\}$, thereby highlighting circuit-dependent feasibility-oriented precision demand. In contrast, Figure 11 reports the minimum-cost operating point under the stochastic workload proxy C=Nm (with runtime used only as a tie-breaker when costs are equal), thereby representing cost-aware adaptive scheduling. Because *N* and *m* jointly determine fidelity, the minimum feasible *m* does not necessarily coincide with the *m* selected by the minimum-cost scheduler. For clarity, Table 7 also reports a representative feasible *N* at $m_{\min}$, chosen as the smallest tested *N* that satisfies the same target-fidelity condition. The observed practical minimum feasible *m* varies substantially across circuits (e.g., c1/c6/c10/c11 at m=64, versus c9 at m=512 for ISC-RE and m=2048 for ISC-PS), indicating circuit-dependent precision demand under a common target-fidelity condition. This trend is also qualitatively consistent with the persistent-sensitivity analysis: circuits with stronger persistent behavior (larger $\gamma_{c}$) often require larger practical *m*, although we do not claim predictive sufficiency from $\gamma_{c}$ alone. The reported $m_{\min}$ values are specific to the chosen target-fidelity condition $F_{\mathrm{target}}=0.9995$ and the evaluated (N,m) search space, and should therefore be interpreted as empirical operating-point requirements rather than universal circuit properties or theoretical lower bounds. These results show that precision elasticity is not limited to offline parameter sweeps. Instead, the proposed ISC framework can be used as a target-fidelity adaptive precision scheduling mechanism: for a prescribed fidelity requirement, the simulator selects a low-cost circuit-dependent stochastic precision setting. We emphasize that this is a software-level stochastic cost proxy study; direct runtime/energy benefit validation should be performed in future hardware-aware implementations.

## 8. Discussion

### 8.1. ISC-PS as a Configurable Stochastic Execution Framework

ISC-PS is best interpreted as a configurable stochastic execution-dynamics framework for quantum circuit simulation. Its behavior is tunable through (N,m), enabling controlled studies of how circuit structure interacts with persistent dependence under unified stochastic settings. This work does not aim to model physical quantum noise or to replace deterministic simulation; instead, it focuses on characterizing execution-induced stochastic effects that arise from persistent reuse. From a framework perspective, this enables direct analysis of uncertainty propagation and correlation-sensitive regimes under unified stochastic execution controls.

The practical value is therefore characterization within the framework: RE/PS separation in sweeps, stable high-*m* tail summaries, and circuit-dependent $\gamma_{c}$ rankings provide direct information about persistent stochastic sensitivity under matched controls. In this role, persistent ISC can be used to (i) compare circuits by sensitivity to retained stochastic correlation, (ii) identify operating regions where persistent tail behavior becomes significant, and (iii) guide execution-parameter selection for subsequent deterministic or hardware-specific follow-up studies.

The target-fidelity adaptive precision scheduling results further strengthen this framework view. They show that stochastic precision can be treated as a schedulable resource in a two-dimensional parameter space, where circuit-dependent (N,m) values are selected to satisfy a common fidelity requirement. The selected operating points are not uniform across workloads, and ISC-PS may require more careful scheduling in some cases due to persistent stochastic propagation sensitivity. Because the present study uses a software-level stochastic cost proxy and the same single-seed evaluation protocol as the main benchmark, future work should evaluate robustness of the selected operating points under larger multi-seed ensembles and hardware-conscious runtime measurements.

The emphasis on small-to-medium-scale workloads is therefore intentional. The goal of this study is correlation analysis rather than large-scale quantum simulation throughput, and the relevant correlation structures already appear in compact circuits with nontrivial depth and multi-qubit interaction patterns. In that sense, the reported trends are driven by circuit structure rather than raw problem size alone.

### 8.2. Circuit Robustness Characterization via $\gamma_{c}$

Under matched (N,m) settings, $\gamma_{c}$ provides an interpretable descriptor of circuit-dependent persistent stochastic sensitivity. Low-$\gamma_{c}$ circuits are comparatively robust under persistent reuse, while high-$\gamma_{c}$ circuits exhibit stronger persistent tail behavior and stronger execution-uncertainty amplification under repeated stochastic reuse. Within the proposed framework, $\gamma_{c}$ serves as a comparative execution descriptor rather than a predictive physical quantity.

The observed dependence on depth and two-qubit interaction descriptors suggests that part of the variation is structurally explainable. This indicates that $\gamma_{c}$ captures, at least in part, accumulated correlation-sensitive propagation effects related to circuit structure, although we do not interpret it as a physical-noise parameter or an entanglement measure. The current characterization remains empirical and benchmark-dependent, and results obtained under reduced “light-mode” settings for heavier circuits should be interpreted with care. In practical terms, circuits with greater depth and denser multi-qubit interaction structure tend to provide more opportunities for the persistent-execution sensitivity summarized by $\gamma_{c}$ to grow. The additional $\gamma_{c}$–covariance-proxy trend is consistent with this view, indicating that larger $\gamma_{c}$ values track stronger correlation accumulation and persistent tail variability rather than an arbitrary fit artifact. This result further supports interpreting $\gamma_{c}$ as an empirical framework-level descriptor of accumulated cross-layer correlation effects.

One possible interpretation of the observed RE/PS performance reversal is that persistent stochastic dependence is not uniformly detrimental. In ISC-RE, stochastic dependence is partially decorrelated between stages through re-encoding, whereas ISC-PS preserves inter-stage stochastic structure across propagation steps. Depending on circuit organization, this persistent correlation may either stabilize or amplify execution-induced variability. For relatively structured or weakly scrambling circuits, correlated stochastic propagation may preserve execution continuity and reduce destructive decorrelation effects. By contrast, circuits with stronger interference sensitivity or covariance amplification may experience increased accumulation of persistent stochastic variability under ISC-PS. This interpretation is consistent with the circuit-dependent trends observed in both Table 6 and the $\gamma_{c}$ analysis.

### 8.3. Hardware Implication of Persistent ISC

Persistent execution is potentially hardware-relevant because it can reduce repeated decode/encode and re-randomization steps in stochastic pipelines. At the same time, persistence introduces cross-layer covariance and potential plateau behavior, so appropriate parameter tuning is necessary. In particular, the tunable parameters (N,m) provide a mechanism for runtime adjustment of accuracy and computational cost, which is difficult to achieve with conventional fixed-point or floating-point hardware.

Possible mitigation strategies include increasing *m*, introducing selective re-randomization checkpoints, and adopting hybrid RE/PS scheduling. These directions are not validated hardware implementations in this work; rather, the present contribution is a characterization of execution regimes that can inform future hardware-oriented design and optimization strategies.

The present study focuses on small- to medium-scale benchmark circuits, and validation on larger circuits remains an important direction for future work. While the observed correlation-sensitive persistent tail behavior is consistent across the evaluated QASMBench workloads, generalization beyond this benchmark set requires further investigation. In practice, the proposed framework is not intended to replace deterministic simulation, but to complement it as a flexible pre-evaluation and design-space exploration tool. By identifying correlation-sensitive circuits early through lightweight stochastic sweeps, persistent ISC can guide the allocation of computational resources toward more detailed deterministic or hardware-aware analysis. A further limitation is that the present evaluation does not use device-calibrated physical noise channels; incorporating such noise models and validating the resulting trends against hardware-oriented measurements are important directions for future work.

## 9. Conclusions

This work presents a precision-elastic persistent stochastic execution framework, ISC-PS, for quantum circuit simulation with explicit and tunable accuracy–cost trade-off. The framework introduces two parameters, (N,m), enabling continuous and runtime-adjustable control of stochastic precision. We show that different execution modes, particularly re-encoded (ISC-RE) and persistent (ISC-PS), exhibit distinct execution behaviors under the same stochastic settings. In persistent execution, circuit-dependent behavior emerges due to accumulated stochastic dependence and uncertainty propagation, which can be summarized using high-*m* descriptors such as ${\mathrm{slope}}_{\mathrm{tail}}$ and $\gamma_{c}$. Across the evaluated benchmarks, these descriptors provide a consistent way to characterize circuit-dependent persistent stochastic sensitivity under the proposed framework, and the observed dependence on circuit structure further indicates that execution behavior is largely driven by interaction patterns and depth rather than arbitrary stochastic variation.

These results support interpreting ISC as an execution-aware computational framework rather than a replacement for deterministic simulation. In particular, the framework enables analysis of circuit-dependent stochastic propagation and accuracy–cost exploration under unified stochastic settings. The target-fidelity scheduling results further show that the two-dimensional ISC precision space can be used to select circuit-dependent operating points that reduce stochastic cost under a prescribed fidelity constraint, suggesting that stochastic precision parameters can be exposed as runtime-configurable scheduling variables rather than static numerical-format choices. In addition, the runtime-adjustable nature of ISC provides a hardware-conscious execution perspective, where computational cost can be adjusted according to resource constraints, making the framework a useful basis for future configurable simulation environments and hardware-oriented implementations.

More broadly, the results suggest that persistent stochastic execution can be treated as a circuit-dependent information-propagation regime, rather than merely as a numerical approximation mechanism. In this view, correlation-sensitive uncertainty propagation, together with compact descriptors such as $\gamma_{c}$, provides a practical basis for analyzing and scheduling adaptive stochastic precision in quantum-circuit simulation workflows.

Future work includes extending the framework to larger-scale circuits, improving structure-aware predictors, and exploring hardware-oriented implementations that leverage the proposed precision–cost trade-off. 

## Figures and Tables

**Table 1:** Qualitative comparison of related approaches and this work.

Method	Arithmetic	Correlation	Cross-Layer	Quantum Sim	Focus
SC correlation studies [10,12]	Stochastic	Explicit	Local	No	Circuit/block design
Quantization/fixed-point (deterministic error propagation) [18,19]	Deterministic	Implicit	Yes	No	Error propagation
Quantum simulation (floating point) [4,5,6]	Deterministic	Implicit	Indirect	Yes	Accuracy vs. speed
This work (ISC-PS)	Stochastic	Explicit	Yes	Yes	Precision-elastic simulation framework

**Table 2:** Key terms, parameters, and descriptors used in the persistent stochastic execution framework.

Term/Symbol	Meaning
Stochastic execution modes	
SC	Stochastic computing based on bitstream statistics.
ISC	Integral stochastic computing with aggregation parameter *m*.
ISC-RE	Re-encoded ISC, where inter-stage dependence is approximately reset between stages.
ISC-PS	Persistent ISC, where dependence is propagated across stages.
Stochastic precision parameters	
*N*	Stochastic bitstream length (sampling depth) per execution setting.
*m*	Number of aggregated stochastic sub-streams.
Quantum circuit parameters	
layer	One gate-application stage (state-update stage) in the simulation flow.
*n*	Number of qubits in the simulated circuit.
*L*	Total number of layers, i.e., circuit depth (total number of gate-application/state-update stages).
#1q gates	Number of single-qubit gates in a circuit.
#2q gates	Number of two-qubit gates in a circuit.
Error/analysis descriptors	
tail regime/high-*m* tail	High-*m* region of error curves used to summarize persistent behavior after transient effects.
$\gamma_{c}$	Circuit-dependent tail descriptor extracted from persistent execution behavior.
$E_{k}$	Theoretical simulation-side error metric relative to the exact statevector at stage *k*.
$e_{k}^{\left(\mathrm{quant}\right)}$	Quantization/clipping-related contribution in the error decomposition.

**Table 3:** Three primary execution modes spanning arithmetic family (SC vs. ISC) and execution handling. Here, N/A indicates that conventional SC has no separate re-encoding or persistent ISC execution-handling mode.

ID	Family	Precision Control	Execution Mode
SC	SC	*N*	N/A
ISC-RE	ISC	(N,m)	Re-Encoded
ISC-PS	ISC	(N,m)	Persistent

**Table 4:** All 12 QASMBench circuits used in the evaluation. Short circuit names are listed for readability, while detailed cross-circuit analysis in the main text highlights representative subsets where appropriate. Abbreviations: QFT, Quantum Fourier Transform; LPN, Learning Parity with Noise; QEC, Quantum Error Correction; QRNG, Quantum Random Number Generator.

Circuit	QASMBench Name	*n*	*L*	#1q Gates	#2q Gates	Notes
c1	cat_state_n4	4	4	1	3	cat state
c2	adder_n4	4	23	13	10	adder
c3	basis_change_n3	3	33	23	10	basis change
c4	qft_n4	4	12	6	6	QFT
c5	lpn_n5	5	11	9	2	LPN
c6	deutsch_n2	2	5	4	1	Deutsch
c7	teleportation_n3_transpiled	3	12	10	2	teleportation
c8	wstate_n3_transpiled	3	35	26	9	W-state
c9	qec_en_n5	5	25	15	10	QEC encoding
c10	qrng_n4	4	4	4	0	QRNG
c11	quantumwalks_n2_transpiled	2	38	35	3	quantum walk
c12	cat_state_n4_transpiled	4	6	3	3	transpiled cat state

**Table 5:** Summary of FP16 trend-consistency metrics at the common operating point (N,m)= (10,000, 1024).

Method/Setting	Pearson	Spearman
Fixed-point baseline (16 fractional bits)	0.940	0.960
ISC-RE (10,000, 1024)	0.628	0.225
ISC-PS (10,000, 1024)	0.703	0.225

**Table 6:** Cross-circuit snapshot at *N* = 10,000 and *m* = 1024. Runtime ratios highlight the efficiency of persistent execution. Here, ${\mathrm{err}}_{*}$ denotes the squared Euclidean error and $t_{*}$ denotes the mean runtime in seconds [s].

Circuit	*n*	*L*	err*_SC_*	err*_RE_*	err*_PS_*	err*_PS_*/err*_RE_*	err*_PS_*/err*_SC_*	*t_SC_* [s]	*t_RE_* [s]	*t_PS_* [s]	*t_PS_*/*t_RE_*	*t_PS_*/*t_SC_*
c1	4	4	$7.380\times 10^{-1}$	$2.411\times 10^{-5}$	$1.419\times 10^{-5}$	0.59	$1.922\times 10^{-5}$	0.509	2.894	0.677	0.234	1.33
c2	4	23	5.026	$1.567\times 10^{-4}$	$3.823\times 10^{-3}$	24.39	$7.606\times 10^{-4}$	2.910	15.85	3.909	0.247	1.34
c3	3	33	1.440	$1.436\times 10^{-4}$	$1.883\times 10^{-3}$	13.11	$1.307\times 10^{-3}$	1.027	5.966	1.420	0.238	1.38
c4	4	12	2.890	$1.281\times 10^{-4}$	$6.514\times 10^{-5}$	0.51	$2.254\times 10^{-5}$	1.515	8.513	2.055	0.241	1.36
c5	5	11	9.144	$1.600\times 10^{-4}$	$8.232\times 10^{-5}$	0.51	$9.003\times 10^{-6}$	6.118	32.82	7.655	0.233	1.25
c6	2	5	$9.554\times 10^{-2}$	$4.553\times 10^{-6}$	$2.661\times 10^{-6}$	0.58	$2.785\times 10^{-5}$	0.0386	0.221	0.0564	0.255	1.46
c7	3	12	$5.084\times 10^{-1}$	$3.638\times 10^{-5}$	$2.501\times 10^{-5}$	0.69	$4.919\times 10^{-5}$	0.371	2.155	0.520	0.241	1.40
c8	3	35	1.548	$1.318\times 10^{-4}$	$6.788\times 10^{-5}$	0.52	$4.384\times 10^{-5}$	1.083	6.172	1.500	0.243	1.38
c9	5	25	15.00	$2.870\times 10^{-4}$	$3.153\times 10^{-3}$	10.99	$2.102\times 10^{-4}$	13.99	74.50	16.48	0.221	1.18
c10	4	4	$7.864\times 10^{-1}$	$3.787\times 10^{-5}$	$1.089\times 10^{-5}$	0.29	$1.385\times 10^{-5}$	0.479	2.742	0.682	0.249	1.42
c11	2	38	$3.526\times 10^{-1}$	$3.990\times 10^{-5}$	$5.976\times 10^{-3}$	149.78	$1.695\times 10^{-2}$	0.283	1.671	0.396	0.237	1.40
c12	4	6	$9.874\times 10^{-1}$	$3.526\times 10^{-5}$	$2.206\times 10^{-5}$	0.63	$2.234\times 10^{-5}$	0.716	4.123	0.996	0.242	1.39

**Table 7:** Empirical minimum feasible multiplicity $m_{\min}\left(c;F_{\mathrm{target}}\right)$ under $F_{\mathrm{target}}=0.9995$. The reported *N* values are representative feasible settings at $m_{\min}$, selected to indicate feasibility at the minimum multiplicity; this feasibility-oriented criterion is distinct from the minimum-cost operating-point selection shown in Figure 11.

Circuit	$m_{\min}$ (ISC-RE)	*N* at $m_{\min}$ (ISC-RE)	$m_{\min}$ (ISC-PS)	*N* at $m_{\min}$ (ISC-PS)
c1	64	10,000	64	20,000
c2	256	20,000	512	10,000
c3	128	20,000	256	10,000
c4	128	10,000	256	10,000
c5	256	20,000	512	10,000
c6	64	5000	64	2000
c7	64	10,000	256	5000
c8	128	20,000	256	10,000
c9	512	20,000	2048	5000
c10	64	5000	64	20,000
c11	64	20,000	64	20,000
c12	64	20,000	128	10,000

**Figure A1:**
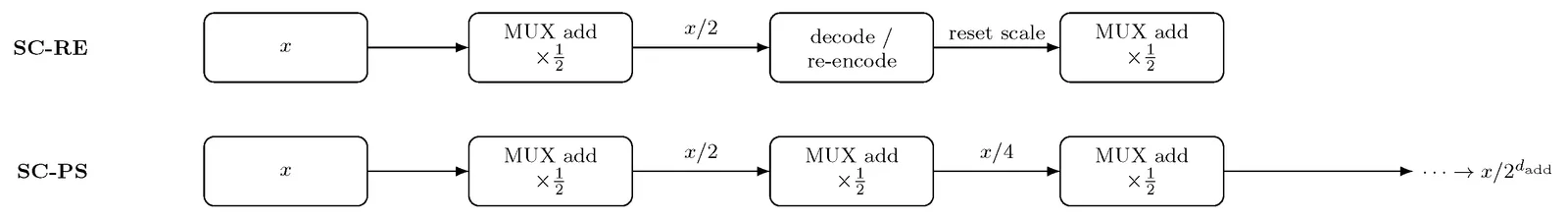
Conceptual comparison between SC-RE and SC-PS. In SC, MUX-based additions introduce an implicit 1/2 scaling per stage. Re-encoding resets this scaling, while persistent execution accumulates it, leading to exponential attenuation with depth. This structural limitation motivates excluding SC-PS from the main framework.

**Figure A2:**
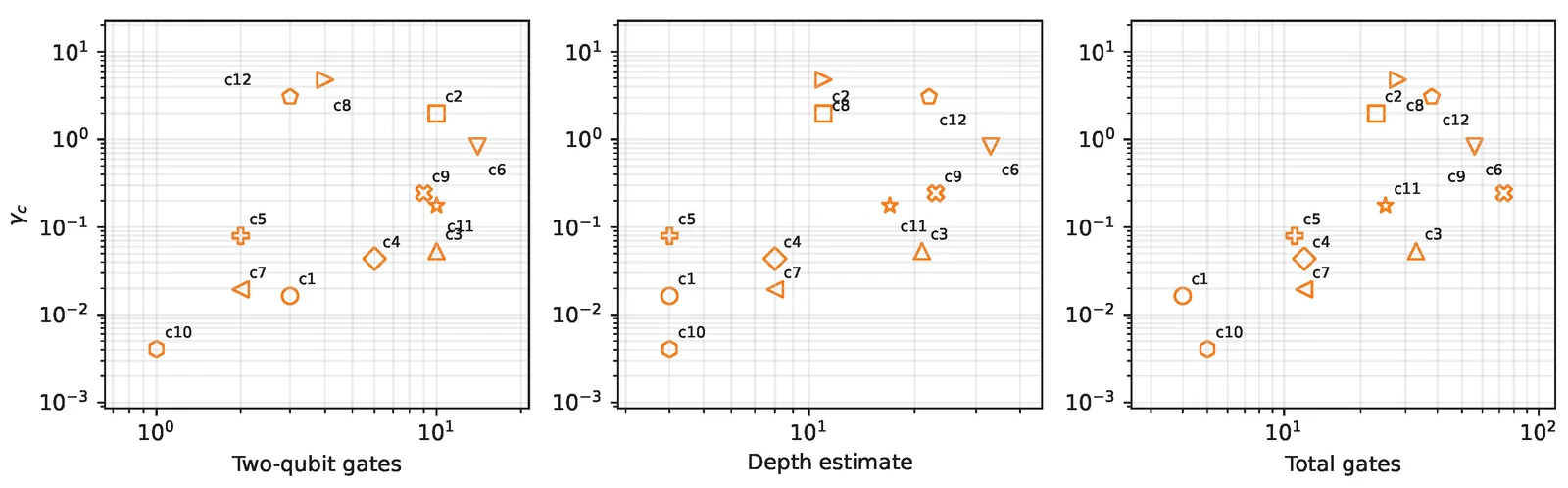
$\gamma_{c}$ versus circuit-structure descriptors for c1–c12. The depth estimate corresponds to circuit depth *L*, and interaction-related descriptors (e.g., number of two-qubit gates) are used as proxies for correlation propagation. The observed trends indicate that deeper and interaction-heavy circuits tend to exhibit stronger persistent amplification.

**Figure A3:**
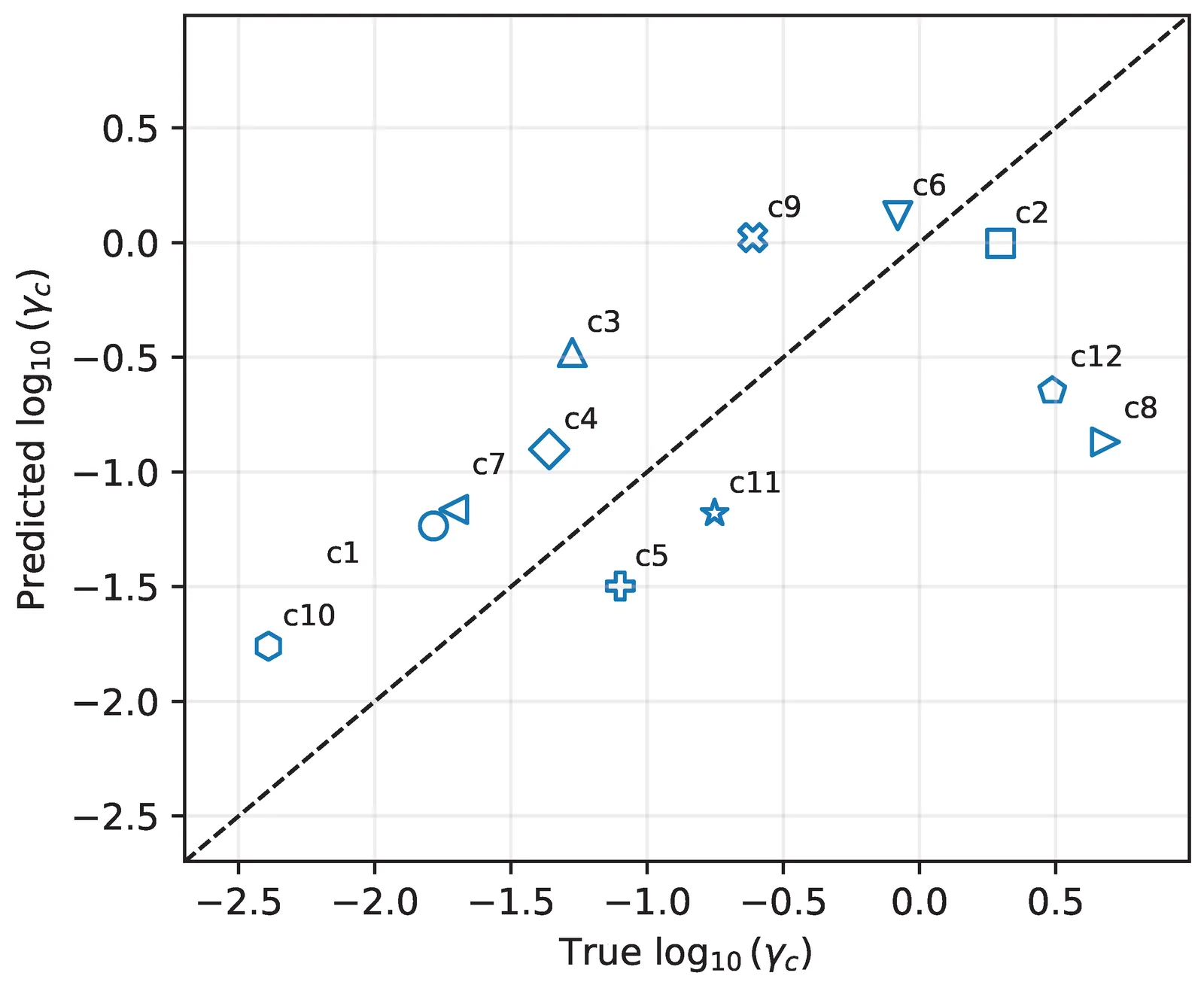
Predicted versus measured (true) $\gamma_{c}$ values for the selected descriptive models. Predicted values are obtained from a simple regression fit based on circuit-structure descriptors. The dashed line denotes the identity line, where predicted and measured $\log_{10}\left(\gamma_{c}\right)$ values are equal. The comparison is intended as exploratory structural-association analysis and screening-oriented description, not as a validated predictor.

**Figure A4:**
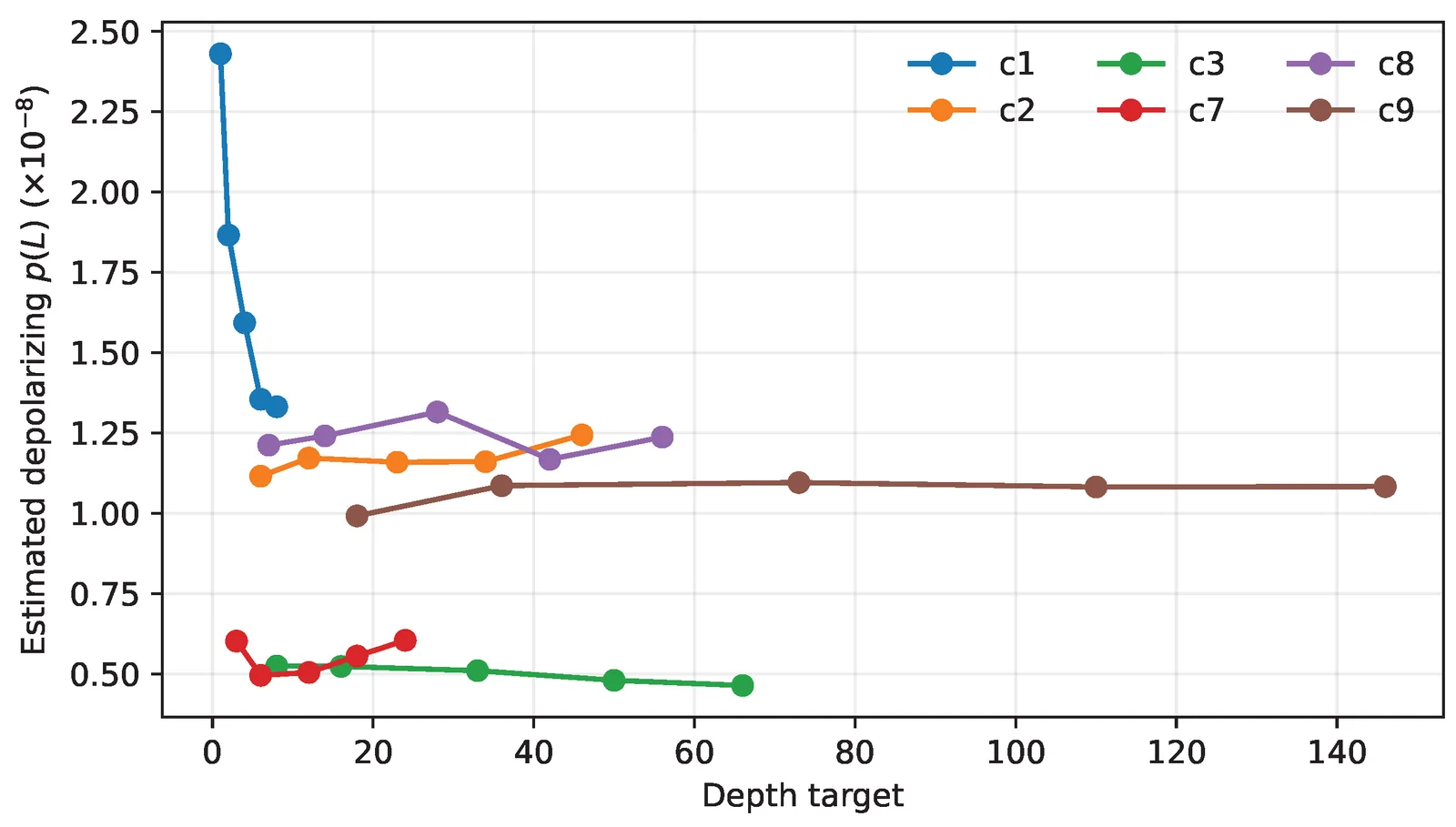
Depth-dependent effective parameter extracted from persistent ISC sweeps. The fitted trend indicates depth-sensitive persistent amplification and motivates its use as a quick structural sensitivity indicator.

## Data Availability

The data and source code presented in this study are openly available at https://github.com/nonizawa/persistent-isc-quantum-sim (accessed on 17 June 2026).

## Appendix A. On the Exclusion of Persistent SC (SC-PS)

This appendix explains why persistent execution is not considered for standard stochastic computing (SC) in this work.

In conventional SC, addition is implemented using a MUX-based scaled adder. This operation effectively computes the average of two inputs, introducing an implicit scaling factor of approximately 1/2 per addition stage. Under re-encoded execution (SC-RE), this scaling effect is mitigated by decoding and re-encoding between stages, which restores numerical range.

In contrast, under persistent execution (SC-PS), this attenuation accumulates across layers. After $d_{\mathrm{add}}$ effective addition stages, the magnitude is reduced approximately as

(A1) $$x\;\to \;\frac{x}{2^{d_{\mathrm{add}}}}.$$

As a result, the dynamic range shrinks exponentially with circuit depth. Maintaining usable precision would therefore require exponentially increasing bitstream length, which is impractical for multi-stage quantum circuit simulation.

This behavior is not a tuning artifact but a structural limitation of SC arithmetic under persistent reuse. In contrast, ISC introduces an integer accumulation mechanism that preserves magnitude without implicit attenuation, making persistent execution (ISC-PS) numerically stable and practically meaningful.

For this reason, SC-PS is excluded from the main analysis, and persistent execution is defined only for ISC in this work. Figure A1 illustrates this attenuation contrast schematically.

## Appendix B. Additional Analyses on Structural Predictors and Depth Sensitivity

We further analyze whether variation in $\gamma_{c}$ can be explained by simple circuit-structure descriptors derived from QASM representations.

In Figure A2, $\gamma_{c}$ is plotted against several structural features, including circuit depth and interaction-related metrics. Here, the depth estimate corresponds to the circuit depth *L*, defined as the total number of gate-application layers in the simulation flow. Additional descriptors include the number of two-qubit gates and related interaction measures, which serve as proxies for correlation propagation opportunities.

Figure A3 compares measured (true) $\gamma_{c}$ values obtained from stochastic simulation with values from a simple regression model based on these structural descriptors. The regression is fit to the observed $\left(\mathrm{structure}\to \gamma_{c}\right)$ relationship across circuits and is used as a descriptive screening model rather than a validated predictor. The agreement trend indicates that a portion of persistent amplification behavior can be explained by circuit structure, although the model is not intended to provide exact prediction.

Interaction-heavy and deeper structures generally align with larger persistent amplification. We interpret this as structural explanatory signal for stochastic execution profiling, not as an exact physical law.

To test depth dependence, we perform a depth-factor sweep and fit an effective depolarizing-like parameter $p_{dep}$ as a compact signature. Figure A4 shows the fitted trend. This is not a claim of physical-channel equivalence; it is a compact effective signature of how persistent amplification scales with depth under fixed stochastic settings.

## References

[1] Nielsen M.A., Chuang I.L. (2010). Quantum Computation and Quantum Information.

[2] Markov I.L., Shi Y. (2008). Simulating quantum computation by contracting tensor networks. SIAM J. Comput..

[3] Vidal G. (2003). Efficient classical simulation of slightly entangled quantum computations. Phys. Rev. Lett..

[4] (2020). Google Quantum AI; Quantumlib Contributors. qsim. High-Performance Quantum Circuit Simulator. https://github.com/quantumlib/qsim.

[5] Qiskit Development Team (2024). Qiskit Aer. High-Performance Quantum Circuit Simulators. https://github.com/Qiskit/qiskit-aer.

[6] NVIDIA (2024). NVIDIA cuQuantum SDK. GPU-Accelerated Quantum Simulation Libraries. https://developer.nvidia.com/cuquantum-sdk.

[7] Li A., Stein S., Krishnamoorthy S., Ang J. (2023). QASMBench: A low-level QASM benchmark suite for NISQ evaluation and simulation. ACM Trans. Quantum Comput..

[8] Lagostina L., Volpe D., Zamboni M., Turvani G. (2025). AEQUAM: Accelerating Quantum Algorithm Validation through FPGA-Based Emulation. IEEE Access.

[9] Liang S., Lu Y., Guo C., Luk W., Kelly P.H.J. PCQ: Parallel Compact Quantum Circuit Simulation. Proceedings of the 32nd IEEE Annual International Symposium on Field-Programmable Custom Computing Machines (FCCM).

[10] Alaghi A., Hayes J.P. (2013). Survey of stochastic computing. ACM Trans. Embed. Comput. Syst..

[11] Ardakani A., Leduc-Primeau F., Onizawa N., Hanyu T., Gross W.J. (2017). VLSI Implementation of Deep Neural Network Using Integral Stochastic Computing. IEEE Trans. Very Large Scale Integr. (VLSI) Syst..

[12] Alaghi A., Ting P., Lee V.T., Hayes J.P., Gross W.J., Gaudet V.C. (2019). Accuracy and Correlation in Stochastic Computing. Stochastic Computing: Techniques and Applications.

[13] Lee V.T., Alaghi A., Ceze L. (2018). Correlation Manipulating Circuits for Stochastic Computing. arXiv.

[14] Wang M., Tannu S., Nair P.J. (2025). Accelerating Simulation of Quantum Circuits under Noise via Computational Reuse. Proceedings of the 52nd Annual International Symposium on Computer Architecture, Tokyo, Japan, 21–25 June 2025.

[15] Grürl T., Kueng R., Fuß J., Wille R. (2020). Stochastic Quantum Circuit Simulation Using Decision Diagrams. arXiv.

[16] Reynolds P.J., Tobochnik J., Gould H. (1990). Diffusion Quantum Monte Carlo. Comput. Phys..

[17] Yang Y., Lu B.N., Li Y. (2021). Accelerated quantum Monte Carlo with mitigated error on noisy quantum computers. arXiv.

[18] Gupta S., Agrawal A., Gopalakrishnan K., Narayanan P. (2015). Deep learning with limited numerical precision. Proceedings of the 32nd International Conference on Machine Learning, Lille, France, 7–9 July 2015.

[19] Micikevicius P., Narang S., Alben J., Diamos G., Elsen E., García D., Ginsburg B., Houston M., Kuchaiev O., Venkatesh G. Mixed precision training. Proceedings of the International Conference on Learning Representations (ICLR).

[20] Han J., Orshansky M. Approximate computing: An emerging paradigm for energy-efficient design. Proceedings of the 2013 18th IEEE European Test Symposium (ETS).

[21] Camsari K.Y., Salahuddin S., Datta S. (2017). Stochastic p-bits for invertible logic. Phys. Rev. X.

[22] Zheng X., Lin D., Chen H. (2026). Grover Adaptive Search-Based Hybrid Benders Decomposition for Mixed-Integer Linear Programs. IEEE Trans. Quantum Eng..

[23] Osca J., Vala J. (2023). SOQCS: A Stochastic Optical Quantum Circuit Simulator. arXiv.

[24] Onizawa N. (2026). Persistent ISC Quantum Simulation. https://github.com/nonizawa/persistent-isc-quantum-sim.

[25] Lukac M., Onizawa N., Nagayama S. A Stochastic-Computing-Based Framework for Quantum Circuit Simulation. Proceedings of the IEEE International Symposium on Multiple-Valued Logic (ISMVL).

